# Lipid complexation reduces rice starch digestibility and boosts short-chain fatty acid production via gut microbiota

**DOI:** 10.1038/s41538-023-00230-1

**Published:** 2023-10-18

**Authors:** Yi Shen, Zengxu An, Zongyao Huyan, Xiaoli Shu, Dianxing Wu, Ning Zhang, Nicoletta Pellegrini, Josep Rubert

**Affiliations:** 1 State Key Laboratory of Rice Biology, Key Laboratory of the Ministry of Agriculture for Nuclear-Agricultural Sciences, Zhejiang University, Hangzhou, 310058 PR China; 2 Food Quality and Design Group, Wageningen University & Research, P. O. Box 17, 6700 AA Wageningen, The Netherlands; 3 Hainan Institute of Zhejiang University, Yazhou Bay Science and Technology City, Yazhou District, Sanya, 572025 PR China; 4 Department of Agricultural, Food, Environmental and Animal Sciences, University of Udine, via Sondrio 2/A, Udine, 33100 Italy

**Keywords:** Metabolomics, Microbiome

## Abstract

In this study, two rice varieties (RS4 and GZ93) with different amylose and lipid contents were studied, and their starch was used to prepare starch-palmitic acid complexes. The RS4 samples showed a significantly higher lipid content in their flour, starch, and complex samples compared to GZ93. The static in vitro digestion highlighted that RS4 samples had significantly lower digestibility than the GZ93 samples. The C_∞_ of the starch-lipid complex samples was found to be 17.7% and 18.5% lower than that of the starch samples in GZ93 and RS4, respectively. The INFOGEST undigested fractions were subsequently used for in vitro colonic fermentation. Short-chain fatty acids (SCFAs) concentrations, mainly acetate, and propionate were significantly higher in starch-lipid complexes compared to native flour or starch samples. Starch-lipid complexes produced a distinctive microbial composition, which resulted in different gene functions, mainly related to pyruvate, fructose, and mannose metabolism. Using Model-based Integration of Metabolite Observations and Species Abundances 2 (MIMOSA2), SCFA production was predicted and associated with the gut microbiota. These results indicated that incorporating lipids into rice starch promotes SCFA production by modulating the gut microbiota selectively.

## Introduction

Rice is a staple food for over half of the population in the world. Rice is mainly composed of starch that can be rapidly digested within the human gastrointestinal tract, increasing blood glucose levels^1^. Therefore, most rice has a high glycemic index (GI). Consuming large amounts of high-GI food could increase the incidence of health issues such as obesity, type II diabetes, and cardiovascular disease^2^. Consequently, it is imperative to explore viable approaches to slow down the rate of starch digestion in foods, including rice. According to the digestion rate and extent, starches can be divided into three classes: rapidly digestible starch, slowly digestible starch, and resistant starch (RS)^3^. RS content can be increased by either “in-plant” breeding rice with high content of RS or processing strategies on rice starch such as complexation with lipids.

On the one hand, for “in-plant” breeding, researchers have created wide high RS rice varieties via chemical mutagenesis, radiation mutagenesis, and genetic engineering in recent years. On the other hand, complexation with ligands such as lipids, proteins, and polyphenols can lower the digestion rate of starch^4^. Lipids such as oil and free fatty acids can be complexed with starch under certain conditions (i.e., 90–100 °C). The hydrophobic chain of lipids can be inserted into the spiral cavity of starch and form a stable structure, which rearranges into undigestible domains^5^. Several studies showed that the complexation of exogenous lipids and starch leads to decreased starch digestibility^4^. In the small intestine, the starch-lipid complexes are not digested by amylases, reaching nearly intact the colonic region^3^. In the lower gastrointestinal tract, the undigestible complex, also called RS type 5 (RSV), is used by the gut microbiota as a substrate^4^.

RS intake modulates the gut microbiota, and the effects of various types of RSs on the composition of microbial communities are different^6^. As a substrate of microbial fermentation, RS significantly influences the gut microbial metabolites, such as increasing the production of short-chain fatty acids (SCFAs), mainly acetate, propionate, and butyrate^7^. Zhou et al.^8^ used an in vitro fermentation model to show that complex starch with saturated fatty acids increased SCFAs level during fermentation. An in vivo experiment showed that mice consuming RS diets had higher butyrate concentrations than control diets^9^. Previous studies showed that dietary RS has potential health benefits, such as reducing the risk of diet-dependent disorders^6^. RSV intake was reported to reduce postprandial glycemic responses and is promising in preventing metabolic syndromes such as type II diabetes^10^. However, the colonic fermentation of RSV, especially derived from rice, and its effect on gut health has been barely studied^11^. Additionally, most studies have examined starch-lipid complexes without taking digestion into account^8,11,12^, exposing the gut microbiota to unreal-life scenarios. Thus, in this study, we comprehensively investigated the in vitro physiological behavior of high-lipid rice RS4, low-lipid rice GZ93, and the palmitic acid-complexed samples, to elucidate the interactions between these samples and the gut microbiota. Samples were first digested in vitro according to the INFOGEST protocol^13^, and consecutively the INFOGEST undigested fractions were employed as a substrate on fecal batch cultures to investigate gut microbiota functionality. By comparing the physiological effect of naturally high-lipid rice (RS4) and processed high-lipid rice (palmitic acid-complexed samples) with low-lipid rice GZ93, we aim to reveal the role of high-lipid rice modulating the gut microbiota.

## Results and discussion

### Proximal compositions and thermal properties of RS4 and GZ93 samples

**Fig. 1:**
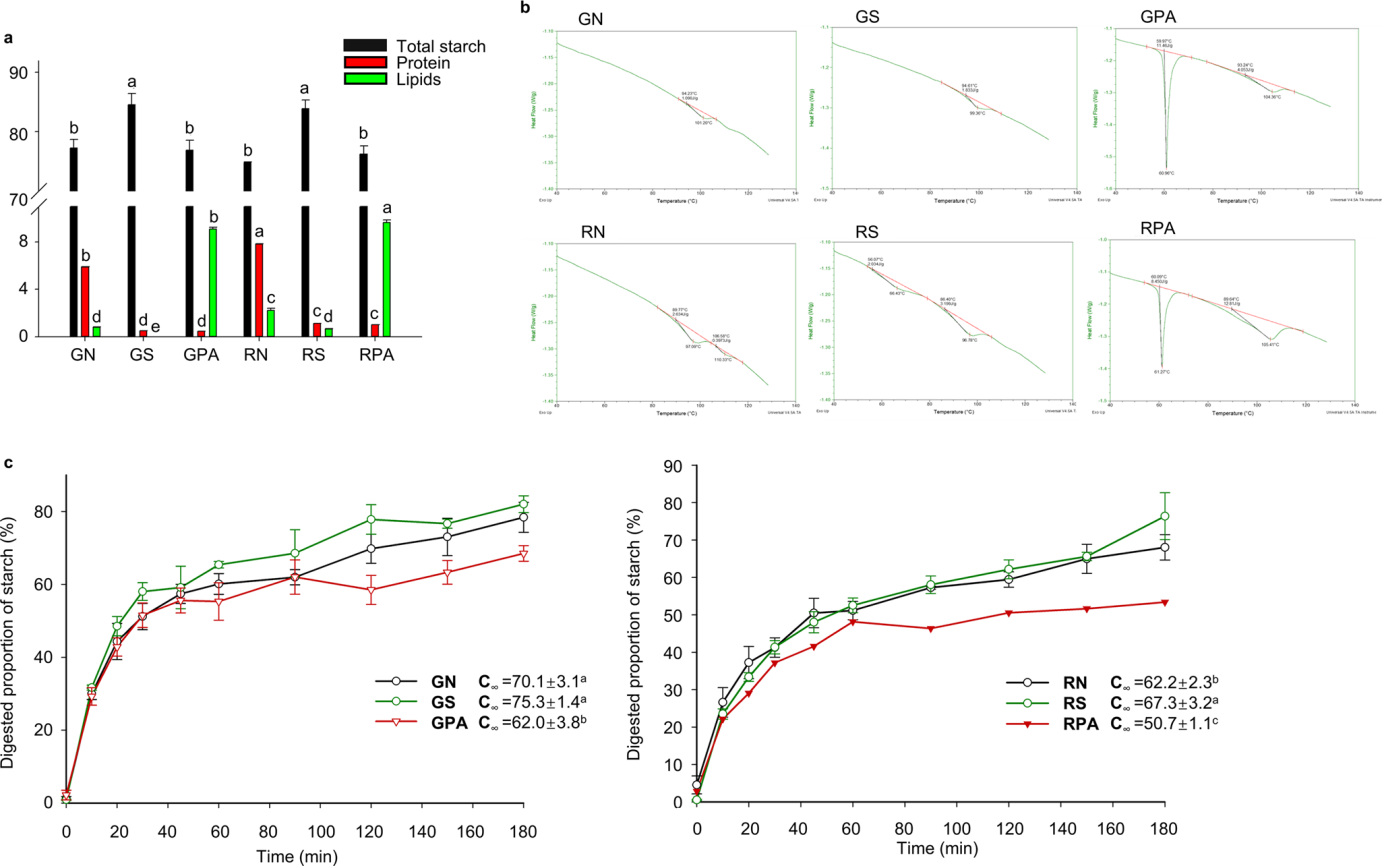
Composition, thermal properties, and digestion properties of analyzed samples. **a** Starch, protein, and lipids content of analyzed samples, (**b**) Thermal properties of analyzed samples, (**c**) In vitro starch digestion properties of analyzed samples. GN: GZ93 native flour, GS: GZ93 starch, GPA: GZ93 starch complexed with palmitic acid, RN: RS4 native flour, RS: RS4 starch, RPA: RS4 starch complexed with palmitic acid, C_∞_: concentration of the percentage of digested starch at infinite time. Different letters indicate significant differences at the 0.05 level. The data are presented with means ± standard deviation. Error bar indicates the standard deviation.

Firstly, we measured the total starch, protein, and lipid content of the samples (Fig. 1a). No significant difference was observed between the total starch contents of RS4 native flour (RN) and GZ93 native flour (GN). However, the protein content (7.82%) and lipid content (2.23%) of RN was significantly higher than that of GN (protein content 5.87%, and lipid content 0.79%). After starch isolation, the total starch contents of RS4 starch (RS) and GZ93 starch (GS) increased by about 9% and 10% compared to those of RN and GN. The protein contents of RS and GS significantly decreased to 1.10% and 0.50%. The lipid content of RS was 0.62%, whereas it was not detected in GS. These data showed that almost all proteins and lipids were removed in isolated starch of RS4 and GZ93. However, when complexed with palmitic acid, the lipid content of RS4 and GZ93 complex (RPA and GPA, respectively) significantly increased to 9.65%, indicating the high efficiency of the complexation method adopted in this study.

Before palmitic acid, commercial rice oil (with triglyceride >80%) was used to complex with RS4 and GZ93 starch to mimic natural starch-endogenous lipid complex. However, the complex was hardly obtained due to the relatively large molecular structure of triglyceride, which served as a large steric hindrance that made it difficult for triglyceride to enter the cavity of starch for complex formation^14,15^. Therefore, this study used the most abundant fatty acid in rice oil (palmitic acid) to complex with starch.

To have more indications of the structure of the starch-lipids complex, the thermal properties of samples were measured using DSC (Fig. 1b). RS displayed two typical endothermic peaks. The second peak (*Tp* 97 °C) was attributed to the melting of amylose-lipids complexes, which was also observed by Zhang et al. (2019)^1^. The melting peak of the starch-fatty acid complex usually appeared at around 94–121 °C^14^. The *Tp* of amylose-lipid complex in RN was 110 °C^16^. After complexation with palmitic acid, RPA had two typical endothermic peaks. The first sharp peak (*Tp* 61 °C) represented the melting of palmitic acid. The second peak was irregular and predicted to be a merged peak of double helical amylose crystallites and amylose-palmitic acid complex^16^, while the latter contributed to the most *ΔH* of the peak with *Tp* at about 105 °C. For this peak, *ΔH* in RPA and RN was 12.1 and 0.4 J/g, respectively, indicating the different amounts of the amylose-palmitic acid complex. Similar results were observed in GZ93 samples. While the lower *ΔH* (3.7 J/g) of GPA, compared to 12.1 J/g of RPA, may be due to the lower amylose content containing lipid ligands in GZ93.

### Digestion properties

To verify the influence of endogenous or exogenous starch-lipid complex on rice starch digestion, this study applied INFOGEST protocol to measure the digestion properties of different samples^13^. As shown in Fig. 1c, after 90 min, the digestion rate of RPA and GPA slowed leading to lower digestion extents than the respective flour and starch samples. GPA had an 11.5% and 17.7% lower C_∞_ than GN and GS, while RPA had a 24.7% and 18.5% lower C_∞_ than RN and RS (Fig. 1c), indicating a high amount of RSV. The C_∞_ of the RN (62.2%) was slightly lower than RS (67.3%), maybe because the endogenous lipid and protein in RS4 flour inhibited starch digestion. Consistent with our results, previous studies also observed decreased starch digestibility after complexation with palmitic acid^17^.

After in vitro digestion, the starch content in the INFOGEST undigested fractions is shown in Supplementary Table 1. The flour and starch samples of RS4 had similar remaining starch content after digestion (0.30 and 0.28 g/g), the flour and starch sample of GZ93 also had similar remaining starch content (0.24 and 0.20 g/g). In contrast, the RPA and GPA had significantly higher remaining starch content (0.42 and 0.32 g/g, respectively). Moreover, the remaining starch content of GPA was lower than RPA but comparable to RN and RS. The digestibility of starch is affected by both the quality and the quantity of the single-helix amylose-lipid complexes^17^. Since RS4 had a higher content of endogenous amylose than GZ93^3^, RPA had more amylose complexed with lipids than GPA. These results suggested that complexation with lipids decreased the digestibility of starch.

### SCFAs production of different samples during batch fermentation

SCFAs are the main end-products of starch fermentation and (bio)indicators of the activity and composition of gut microbiota^11^. SCFAs have several physiological functions, such as mediating colon pH to inhibit the growth of certain pathogenic bacteria, supplying energy to gut microbiota and intestinal epithelial cells, and sustaining the barrier function of the intestinal mucosa^7,18,19^. The higher content of SCFAs produced from high-lipid rice RS4 samples compared to GZ93 samples can potentially improve gut health.

**Fig. 2:**
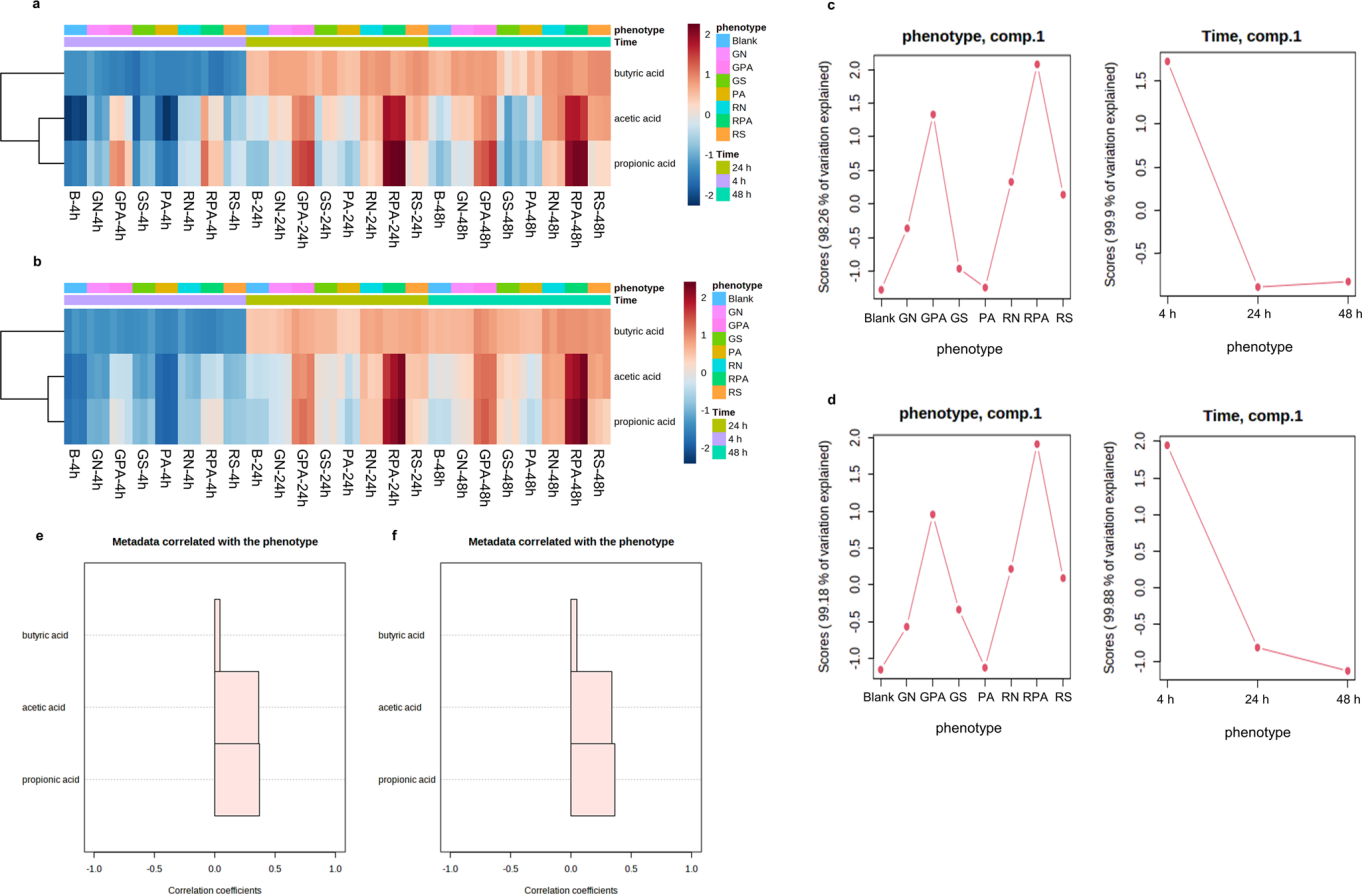
The data matrix of SCFA production of analyzed samples processed by MetaboAnalyst 5.0. Heatmap showing similarities and patterns between different samples in donor1 (**a**) and donor2 (**b**); ANOVA Simultaneous Component Analysis (ASCA) of two main variables explaining to variation in donor1 (**c**) and donor2 (**d**); Correlation analysis of short-chain fatty acids content with phenotype in donor1 (**e**) and donor2 (**f**). GN: GZ93 native flour, GS: GZ93 starch, GPA: GZ93 starch complexed with palmitic acid, RN: RS4 native flour, RS: RS4 starch, RPA: RS4 starch complexed with palmitic acid.

The in vitro digested materials (blank, palmitic acid, native flour, starch, and starch-lipid complexes) were fermented and slurries were collected at 4 h, 24 h, and 48 h to measure SCFAs concentrations. The 3D PCA (Supplementary Fig. 1a, b) showed that palmitic acid and blank samples were almost overlapped at all three-time points indicating that the addition of palmitic acid did not affect SCFAs production. However, starch-lipid complexes showed distinct clustering compared to other sample types, which indicated that complexation with lipids significantly changed the fermentation, especially the SCFAs production. RPA samples differed from GPA samples. Samples at 4 h were separated, while samples at 24 h and 48 h were overlaid, which means samples at these two-time points had close SCFAs production. Figure 2a, b showed that acetic and propionic acid concentrations had closer clustering. Acetic and propionic acids of starch-lipid complexes had significantly higher concentrations than other samples. In contrast, butyric acid concentration didn’t show a significant difference between starch-lipid complexes and flour/starch samples. Moreover, RS and RN samples showed higher abundances of acetic and propionic acids than GS and GN samples for all time points, which may be due to the higher remaining starch residues of RS and RN samples, indicating the high lipid & amylose variety RS4 had a better fermentation property in terms of SCFAs production. ASCA showed the ‘Phenotype’ and ‘Time’ factors effects. There were 8 components of the factor ‘Phenotype’ and 3 components of the factor ‘Time’ (Supplementary Fig. 1c, d). According to the scores of the first component, which explained more than 98% of the variation on the factor ‘Phenotype’ (Fig. 2c, d), two starch-lipid complexes had significantly higher scores than other samples. Furthermore, each type of sample from the RS4 variety (RPA, RN, and RS) showed higher scores than the same type of samples from the GZ93 variety (GPA, GN, and GS). The first component of the factor ‘Time’ explained more than 99.8% of the variation, and 4 h fermentation samples showed significantly different scores compared to 24 h and 48 h fermentation samples. Correlation analysis showed that propionic acid and acetic acid content had a much higher correlation with the factor ‘phenotype’ than the content of butyric acid (Fig. 2e, f). These results indicated that the breeding effect (high-lipid and amylose rice variety - RS4) and processing effect (complexing with lipids) could lead to higher production of SCFAs.

**Fig. 3:**
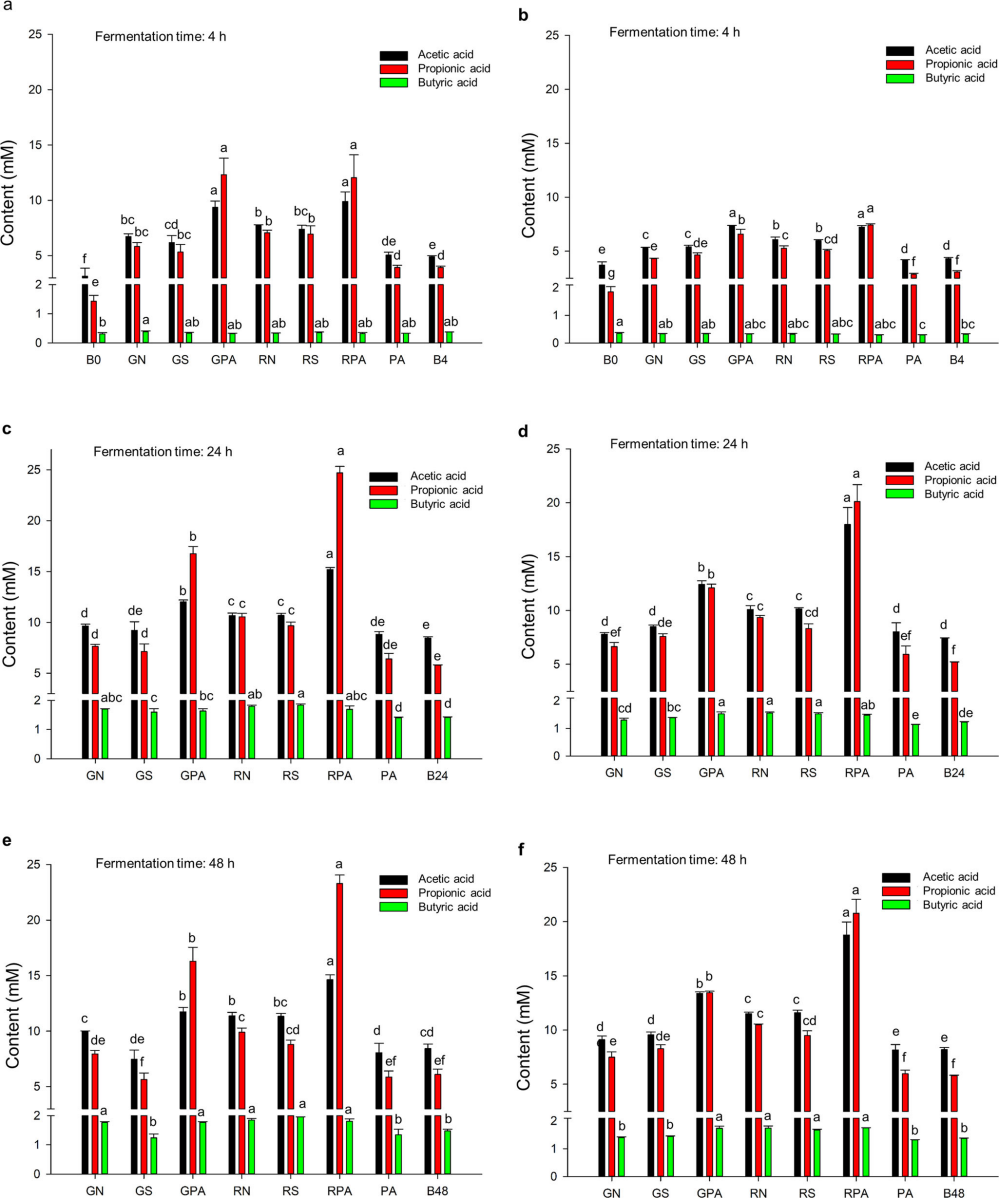
Produced SCFAs of analyzed samples. **a**, **c**, **e** SCFAs produced by samples of donor1 when fermented for 4 h, 24 h, 48 h, (**b**), (**d**), (**f**) SCFAs produced by samples of donor2 when fermented for 4 h, 24 h, 48 h. RN: RS4 native flour; RS: RS4 starch; RPA: RS4 starch complexed with palmitic acid; GN: GZ93 native flour; GS: GZ93 starch; GPA: GZ93 starch complexed with palmitic acid; PA, palmitic acid samples; B0, B4, B24, B48: blank sample fermented at 0, 4, 24, 48 h. Different letters indicate significant differences at the 0.05 level. Error bar indicates the standard deviation.

Figure 3 and Supplementary Table 2 show the SCFAs production of analyzed samples at 3-time points. Previous research works have used native starch-lipid complexes on fecal batch cultures, obtaining relatively high SCFA concentrations due to excess substrate^8,11,12^. This study used INFOGEST undigested fractions containing fewer starch residues, recapitulating a real-life scenario. GPA and RPA produced significantly more acetic and propionic acids than their respective flour and starch samples at all time points (Fig. 3 and Supplementary Table 2). After 4 h fermentation, butyric acid production showed no difference in the analyzed samples, and no significant differences were observed in SCFAs production between GZ93 and RS4 samples (Fig. 3a, b).

After 24 h fermentation, in donor1. RN and RS produced significantly more acetic acid and propionic acid after fermentation than GN and GS (except propionic acid content of starch samples in donor 2), which may be due to the higher content of RS in RS4 rice (Fig. 3c, d). Moreover, RPA had 15.19 mM of acetic acid concentration and 24.71 mM of propionic acid concentration, significantly higher than the acetic and propionic acid concentrations of 12.03 mM and 16.76 mM in GPA. Unlike 4 h, after 24 h fermentation, the butyric acid content of RS (1.83 mM) was significantly higher than it of GS (1.60 mM) in donor1, while in donor2, RN and RS both had a significantly higher content of butyric acid than GN and GS.

After 48 h fermentation, each sample had similar SCFA production to that after 24 h fermentation, indicating that microorganisms fully utilized the remaining starch after 24 h fermentation (Fig. 3e, f). Notably, only palmitic acid-complexed samples produced higher levels of propionic acid than acetic acid after 48 h fermentation. It can be assumed that propionic acid is a characteristic fermentation product of the rice starch-palmitic acid complex. After 48 h fermentation, acetic acid and propionic acid were the leading products and had a proportion over 70% of the total SCFAs.

Qin et al.^11^ reported that the production of SCFAs reached the maximum after 12 h fermentation. The total content of acetic and propionic acids was about 76% of all SCFAs after 24 h fermentation. These conclusions are consistent with our results, except that acetic acid was the dominant product in the research of Qin et al.^11^. Zhou et al.^8^ claimed that the anaerobe could not utilize saturated fatty acids in the colon. However, it could boost fermentation after complexing with high-amylose maize starch, resulting in more acetic acid, propionic acid, and total SCFA production than high-amylose maize starch alone or debranched starch. The propionic acid and butyric acid production pathways are conserved and substrate-specific, while the production pathways of acetic acid are widely distributed among bacterial groups^20^. Duncan et al.^21^ reported that reduced intake of carbohydrates decreased the proportion of butyric acid to total SCFA in feces. Starch is an essential substrate for lactate production, and butyrate is the main product of lactate fermentation by human intestinal microbiota^22^. When the carbon substrate is limited, gut microbiota may decarboxylate the intermediate product succinate to propionic acid, which regenerates CO_2_ to convert C3 acid to C4 acid (succinate pathway)^22^. The proportion of propionic has been considered beneficial in preventing obesity and type II diabetes^23^. Thus, the higher percentage of propionic acid produced by RSV fermentation may benefit human health.

### Gut microbiota diversity and composition of analyzed fermented samples

#### Bacterial α-diversity and β-diversity

SCFAs are one of the major end-products of the fermentation of dietary fibers by the gut microbiota^22^. The difference in SCFA production may be caused by microbial diversity. To study the effects of RSV on the gut microbiota composition and the cross-linking between SCFAs and microorganisms, the microbial communities of samples after 48 h fermentation were researched by 16S rRNA gene sequencing. The α-diversity indexes were calculated to comprehensively determine the richness and evenness of the community (Supplementary Table 3). Compared to zero time, the feature sequences and Faith index of the blank sample after fermentation increased, indicating the increased bacterial diversity. GPA and RPA had lower feature sequence numbers, and Shannon and Evenness index than the corresponding flour or starch samples. Still, no difference in Faith index was observed, indicating a lower level of bacterial diversity and community evenness of starch-lipid complexes but comparable community richness, mainly caused by forming a specific dominant bacterial community in starch-lipid complexes. This result showed that RSV reduced the gut microbial diversity while increasing certain types of microbial taxa. Zhou et al.^8^ and Qin et al.^11^ also reported that RSV caused significantly lower bacterial diversity than blank samples after in vitro fermentation. Deehan et al.^24^ conducted a study in healthy humans and showed that a higher dose of RS4 increased interindividual variation and reduced community evenness of fecal bacteria. Overall, these results are consistent with our study, which indicates that the complexed starch with palmitic acid can reduce the α-diversity of microbial communities.

**Fig. 4:**
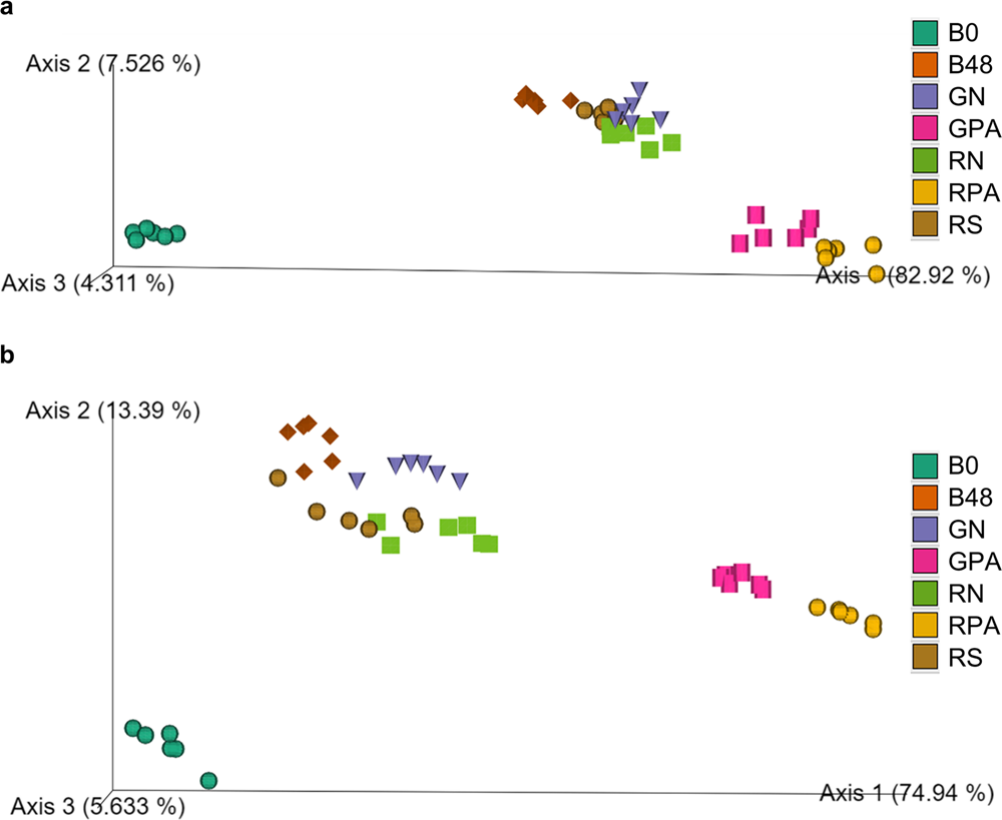
β diversity PCoA on the gut microbiota among different fermented samples. **a** donor1, (**b**) donor2. B0: blank sample at 0 h; GN: GZ93 native flour; GPA: GZ93 starch complexed with palmitic acid; RN: RS4 native flour; RS: RS4 starch; RPA: RS4 starch complexed with palmitic acid; B48: blank sample at 48 h.

In order to identify the distinct variations in microbial community composition between samples, we calculated the β-diversity. The first three principal coordinates presented over 90% of the bacterial community variation (Fig. 4). The blank sample at zero time was different from other samples. After 48 h fermentation, the flour and starch samples of both RS4 or GZ93 samples had similar microbial composition to the blank sample. The gut microbiota composition of GPA and RPA samples were similar, as the dots almost coincided but differed from other samples. These results showed that the fermentation of RSV had a specific effect on the gut microbiota community, reducing the α-diversity and changing the microbial community’s specific composition. Moreover, the distance between RS4 starch-lipid complexes and flour/starch samples was farther than that between GZ93 starch-lipid complexes and flour/starch samples, which showed more variation of β-diversity produced by RPA than GPA. This is possibly owed to more remaining starch in RPA than GPA after digestion or the more starch-lipid complex in RPA than GPA (Fig. 1b). Interestingly, GPA had similar remaining starch content after digestion to that of RN and RS but had significantly different β-diversity after fermentation (Supplementary Table 1, Fig. 4). In contrast, the remaining starch contents between GPA and RPA were significantly different, but their β-diversity was much closer (Supplementary Table 1, Fig. 4). Therefore, the variation of microbial composition among different samples was mainly driven by the content of starch-lipid complex in samples instead of the remaining starch content.

#### The gut microbiota composition

**Fig. 5:**
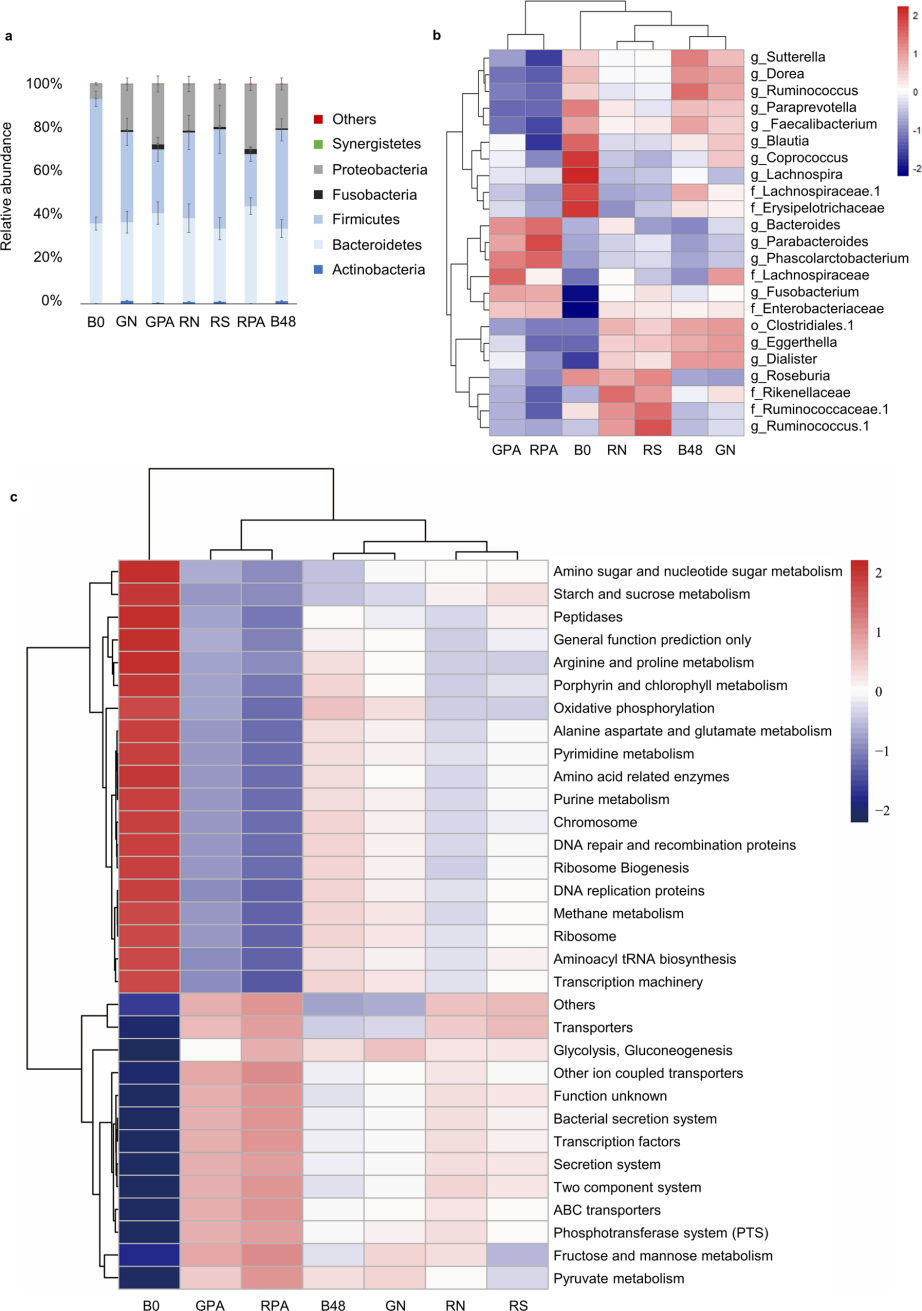
The composition difference of the gut microbiota and the PICRUSt-predicted KEGG ortholog (KO) functions of analyzed fermented samples. **a** The composition difference of the gut microbiota at the phylum level, (**b**) The heatmap of compositional variance of the gut microbiota at the genera level, (**c**) Heatmap showing the PICRUSt-predicted KEGG ortholog (KO) functions of analyzed fermented samples (feces comes from donor 2). Red color indicates relatively high values, whereas blue color means low values. B0: blank sample at 0 h; GN: GZ93 native flour; GPA: GZ93 starch complexed with palmitic acid; RN: RS4 native flour; RS: RS4 starch; RPA: RS4 starch complexed with palmitic acid; B48: blank sample at 48 h. Error bar indicates the standard deviation.

By aligning the feature sequence of each sample with the Greengenes database, phylum and genus level abundance of different substrates are shown in Fig. 5a, b. At the phylum level, *Firmicutes*, *Bacteroidetes*, and *Proteobacteria* were dominant (Fig. 5a). Compared to the flour or starch samples, after fermentation, GPA and RPA had a higher abundance of *Bacteroidetes* and *Proteobacteria* and lowered abundance of *Firmicutes*, which is consistent with the result of Qin et al.^11^. In addition, the starch-lipid complexes had a higher level of *Fusobacteria* than the flour and starch samples. *Firmicutes* can boost metabolizing plant polysaccharides into SCFAs, *Bacteroidetes*, and *Proteobacteria* play essential roles in organic degradation and carbon cycling. At the same time, *Fusobacteria* is associated with the degradation of soluble starch and amylopectin^25–27^. These results suggested that the fermentation of starch-lipid complex influenced gut microbiota composition related to polysaccharide degradation.

At the genus level, GPA and RPA had similar gut microbiota composition. They were classified into one cluster, while all flour or starch samples had similar gut microbiota composition to blank samples after fermentation for 48 h and were classified into another cluster (Fig. 5b). Compared to the other samples, the starch-lipid complexes had a higher abundance of Bacteroides, Parabacteroides, Phascolarctobacterium, Fusobacteriaceae, and Enterobacteriaceae. In comparison, a lower abundance of *Sutterella*, *Dorea*, *Ruminococcus*, *Paraprevotella*, *Faecalibacterium*, *Clostridium*, *Eggerthella*, *Dialister* and *Rikenellaceae* was observed. Among the taxa with higher abundance, *Bacteroides* and *Parabacteroides* are associated with acetate and propionate production^19^. The lower abundance of *Faecalibacterium* and *Clostridium* is related to butyrate production^19^. These results showed that the fermentation of RSV had specific effects on the gut microbiota, such as the increase of some propionate producers, which were related to the metabolic function of the gut microbiota.

#### The predicted gene functional difference of fermented samples

Based on the prediction by PICRUSTs on 16S rRNA amplicon sequences, the metabolic functions between GPA and RPA were similar but differed significantly from other samples (Fig. 5c). Compared to other samples, the gut microbiota of starch-lipid complexes had significantly higher gene expression levels of fructose and mannose metabolism, pyruvate metabolism, etc. Pyruvate metabolism is important for SCFA production, considering that pyruvate is the primary precursor of SCFAs^22^. After pyruvate is produced, the gut microbiota can catabolize pyruvate into lactate, succinate, and acetyl-CoA and finally, these intermediates will be metabolized into SCFAs^19^. On the other hand, propionate can be produced from phosphoenolpyruvate via the succinate pathway or the acrylate pathway from lactate^28^, which are both closely related to pyruvate metabolism. At the same time, significantly lower gene expression levels of glutamic acid and aspartic acid metabolism, DNA repair and recombination protein, etc. were identified. Regarding starch and energy metabolism, GPA and RPA had significantly higher gene expression levels of fructose and mannose metabolism while lower levels of starch and sucrose metabolism than other samples. This can be explained by the lower abundance of *Firmicutes* (Fig. 5a) contributing to metabolizing plant polysaccharides^25^ while the higher abundance of *Bacteroidetes* and *Proteobacteria* which played important roles in carbon cycling and degradation of organic matter^26^ in complex samples.

As Fig. 5 showed, GN and the blank sample had similar gut microbiota composition, predicted functions after 48 h fermentation, and belonged to the same cluster. In contrast, RN and RS belonged to another cluster. The difference may be because of the higher RS content in RS4 samples. Specifically, the abundance of *Parabacteroides, Phascolarctobacterium, Roseburia, Rikenellaceae, Ruminococcus*, and microbial communities related to starch, sucrose metabolism, and bacterial secretion metabolism was higher in RN and RS compared to GN. Compared to RS, RN is more similar to the starch-lipid complexes in the bacterial β diversity and the predicted functions (Fig. 5b, c). The difference is not remarkable, which may be because the amount of endogenous RSV in RS4 samples was not enough to cause significant changes in the microbial composition during fermentation. This result indicated that GPA and RPA had similar KEGG KO function enrichment after fermentation related to the distinctive gut microbiota composition of starch-lipid complexes caused by RSV content, and influenced the SCFA production.

### The association of SCFAs concentration and gut microbiota

**Fig. 6:**
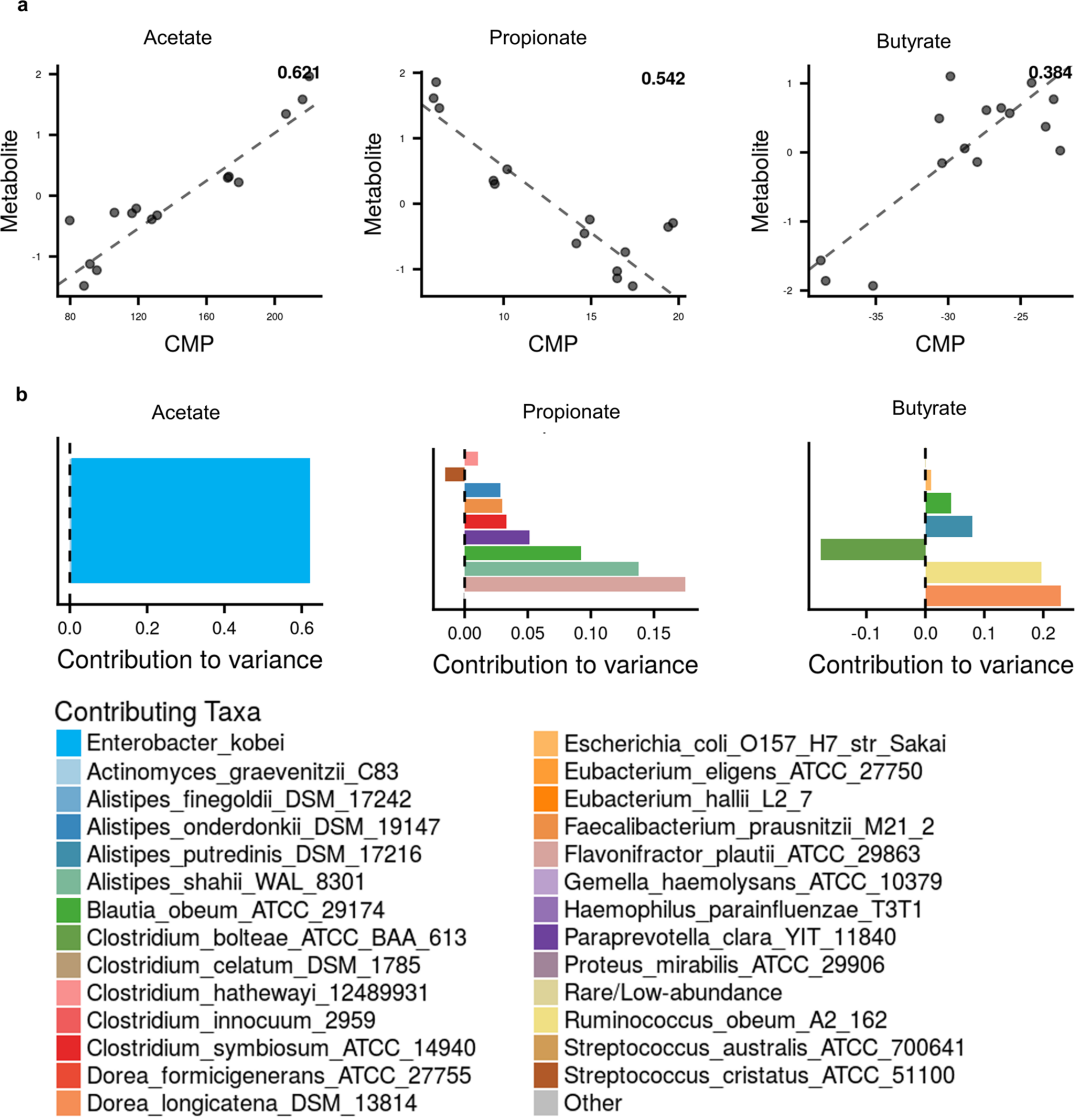
Microbial contribution to SCFAs variance based on MIMOSA2. **a** Comparison plots showed the prediction of community-level metabolic potential scores (CMP) on variation in acetate (according to EMBL_GEMs genomes and models), propionate, and butyrate (according to AGORA genomes and models) levels. The number at the top-right corner of the plot indicated the R-squared of the regression model. **b** Contribution plots showed the contributions of taxa explaining variation in each SCFA; the left of the dotted line represents negative contribution; the right of the dotted line represents positive contribution.

To link the variation of SCFAs concentration with gut microbial composition, we compared total community-level metabolic potential (CMP) scores with SCFAs measurements across GPA, RPA, GN, RN, and RS. We used a regression model to assess whether CMP scores significantly predict SCFA levels, then identified specific taxa that explain variation in each SCFA on the MIMOSA2 website^29^. Well-predicted SCFAs were identified by the CMP score model in all sample groups by a model using a local FDR *q-value* < 0.01 as the significance threshold^30^. The gut microbiota contributed to the changes in the abundance of all three SCFAs: acetate, propionate, and butyrate (Fig. 6a, Supplementary Table 4). The mapped taxa data of all samples are shown in Supplementary Table 5. It is worth mentioning that we compared the AGORA database and EMBL_GEMs database for metabolic model settings, and the CMP prediction of acetate had the highest R-squared when it was based on the EMBL_GEMs database, while the CMP prediction of propionate and butyrate had the highest R-squared when they were based on AGORA database. These differences are possible because the AGORA database is a collection of genome-scale metabolic models of gut microbiota^31^. In contrast, the EMBL_GEMs database is a collection of genome-scale metabolic models for all 5587 reference and representative bacterial genomes in RefSeq^32^, which caused the different numbers and species of taxa that can be mapped to these two models. The comparison plots of acetate and butyrate had a positive slope. By contrast, CMP negatively predicted the propionate. In general, the positive correlation between CMP scores and metabolite levels is considered that the metabolite is putatively microbiome-governed, such as acetate and butyrate^32^. As Fig. 6b showed, *Enterobacter kobei* was predicted as the main contributor to the variation of acetate. It can express *CITACt* gene responsible for acetate transport (Table S4). Moreover, *Enterobacter kobei* belongs to the *Enterobacteriaceae*, and the abundance in starch-lipid complexes was higher than in flour and starch samples (Fig. 5b), which possibly caused the higher concentration of acetate in starch-lipid complexes (Fig. 3f). *Flavonifractor plautii ATCC 29863, Alistipes shahii WAL 8301*, etc. were the main microorganisms causing the variation of propionate level, which explained more than 98% of the total variance (Supplementary Table 4). These microorganisms increase propionate production by expressing the *PPAt2* gene responsible for propionate transport. In this study, these microorganisms had significantly lower abundances in starch-lipid complexes compared to flour and starch samples (Supplementary Table 5). They should cause lower production of propionate in starch-lipid complexes. Although, the results were the opposite (Fig. 3f) and showed a negative correlation between propionate and CMP (Fig. 6a). Other taxa, such as *Streptococcus cristatus ATCC 51100* had a negative contribution to the propionate variation, indicating that it might compensate for or mitigate the predicted metabolic effects of other taxa^33^. In this study, the propionate level showed a negative but strong (R-squared of the regression model was 0.542) association suggesting that propionate was microbiome-governed^29^. This negative but strong association can occur for several reasons. For example, the AGORA model used only one propionate synthesis gene, ‘*PPAt2*’, and one degradation gene, ‘*PPAt2i*’, distributed across 28 different taxa (Supplementary Table 4), suggesting that custom management of associated genes and reaction annotations may improve the model^29^. The negative association also indicates that the significantly higher propionate content in starch-lipid complexes could inhibit the growth of those taxa which are related to propionate synthesis^30^. *Flavonifractor plautii ATCC 29863*, *Blautia obeum ATCC 29174*, *Clostridium symbiosum ATCC 14940* and *Faecalibacterium prausnitzii M21/2* all belong to *Firmicutes* phylum. Li et al.^14^ reported that dietary sodium propionate decreased the abundance of *Firmicutes*^34^, which proved our foregoing results. For the butyrate level, *Dorea longicatena DSM 13814*, *Ruminococcus obeum A2 162*, and *Alistipes putredinis DSM 17216* were the central microorganisms causing the variation of butyrate level. These three microorganisms expressed the *BUTt2* gene responsible for butyrate transport (Supplementary Table 4). The abundance of these three microorganisms was significantly lower in GPA, RPA, RN and RS than in the GN (Supplementary Table 5). Thus, it makes less degradation and more butyrate accumulation in GPA, RPA, RN and RS samples compared to the GN (Fig. 3f). Meanwhile, *Clostridium bolteae ATCC BAA 613* made a substantial negative contribution to the variation of butyrate level with *BUTt2* gene expression and had significantly higher abundance in two starch-lipid complexes than other samples (Supplementary Table 5), which mitigated the predicted metabolic effects of other taxa. Thus, CMP predicted acetate, propionate, and butyrate, and we hypothesized that RSV modulated specific microorganisms and promoted SCFA production.

The present study, which mimicked human digestion and colonic fermentation in vitro, showed that RSV obtained by complexing rice starch with palmitic acid decreased the in vitro digestibility of starch. When the INFOGEST undigested fraction was exposed to the gut microbiota, certain microbial taxa were boosted changing the specific functions to degrade the matrix. During the microbial fermentation, the palmitic acid complexed sample increased SCFA production compared to native flour or starch samples, and SCFA concentrations were strongly associated with specific taxa.

RS4 samples produced higher concentrations of acetic and propionic acids, indicating a better fermentation effect of the high-lipid rice. These results suggest that high-lipid rice, whether naturally or through processing strategies, may potentially have more health effects than low-lipid rice. Further in vitro and in vivo studies are needed to investigate the mechanism of how the starch-lipid complex interacts with the gut microbiota and intestinal epithelial cells.

## Methods

### Materials and reagents

Pepsin (P6887), α-amylase (A3176), pancreatin (P1750, 4×USP specifications), amyloglucosidase (10113), and palmitic acid were purchased from Sigma-Aldrich (Merck KGaA, Germany). KCl, KH_2_PO_4_, NaCl, MgCl_2_·(H_2_O)_6_, CaCl_2_·(H_2_O)_2,_ and pure ethanol were purchased from VWR International B.V. (the Netherlands). KH_2_PO_4_, NaCl, (NH_4_)_2_CO_3_, NaOH, and HCl, as well as the yeast extract, peptone, mucine, and L-cysteine HCl were purchased from Sigma-Aldrich Chemie B.V. (the Netherlands). All the chemicals used are of analytical and chromatographic grade.

### Rice and pre-sample treatment

This study used two rice varieties: RS4 (apparent amylose content 44.7%) and GZ93 (apparent amylose content 16.4%). They were derived from gamma irradiation (300 Gy) of R7954 as previously described^3^. Briefly, dry grains of R7954 were treated by 300 Gy 60-Cobalt gamma rays’ irradiation. Mutants with different endosperm lipid traits were selected from the M2 generation and verified in the subsequent M3–6 generations. The highly advanced generation mutants with stable endosperm lipid contents were chosen for the current study.

The two varieties were grown in Hainan (110°2'1“E, 18°31'36“N), China in 2019. The two varieties were harvested, dried, and stored at 4 °C in darkness. Before analysis, grains were dehulled using a paddy husker (Satake Co., Tokyo, Japan). Then the outer layer (15% of total weight) of brown rice was removed using a rice whitener machine (Satake Corp., Tokyo, Japan) to get white rice. White rice was milled using a cryogenic grinder (6875D, Metuchen, USA) and sieved through 90–160 µm mesh to obtain the rice flour.

Starch was isolated based on the method reported by Lumdubwong & Seib^35^ with modifications. Briefly, 6 g of rice were weighed into a 50 mL centrifuge tube, then 30 mL of 0.2% sodium hydroxide solution and a glass bead were added. The tubes were kept at room temperature with shaking for 3 h. Then, the mixture was centrifuged at 4000 *g* for 20 min. After centrifuge, the supernatant was discarded and the upper layer of the sediment was carefully removed. Then, the sediment was washed with distilled water for three times and the upper layer was removed each time after washing. During the last washing, the pH value of starch suspension was adjusted to 7 and the final sediment was freeze-dried. The starch was milled by a ball miller and sieved through 125 µm mesh for further analysis.

### Measurement of starch, protein, and lipid content

Total starch content was measured using the Total Starch Assay Kit (AA/AMG) (Megazyme Inc., Bray, Ireland). Protein content was measured by Dumas (Thermo Quest NA 2100 Nitrogen and Protein Analyser, Interscience, Breda, the Netherlands) using a protein-to-nitrogen conversion factor of 6.25. D-Methionine was used as a standard. Lipids content was measured using the method reported by Zhang et al.^1^.

### Preparation of starch-palmitic acid complex

The starch-palmitic acid complex was produced using the method reported by Soong et al.^17^ with modifications. Briefly, 0.5 g starch was mixed with 7.5 mL of water in a 50 mL centrifuge tube, then 0.05 g palmitic acid was added. The mixture was vortexed for 2 min for full dispersion. Then the mixture was incubated in boiling water for 30 min, immediately shaking every 5 min. After cooking, the tubes were cooled down at room temperature for 1 h to allow the retrogradation of starch and the formation of the complex.

Rice flour and rice starch samples were cooked in the same way without palmitic acid added. The prepared samples were immediately used for digestion, or freeze dried for thermal property measurement.

### Differential scanning calorimetry (DSC)

The thermal properties of the samples were measured with a differential scanning calorimeter DSC Q200 (TA Instruments, New Castle, USA), according to Shen et al.^3^. The prepared samples were freeze-dried and milled. For each sample, 2.5 mg was weighed in an aluminum DSC pan, then 7.5 µL of deionized water was added. After hermetically sealed, the pans were equilibrated at room temperature for 12 h. Then the sample pans were scanned at a heating rate of 4 °C/min from 30 to 130 °C with a hermetically sealed empty pan as a reference. The onset temperature (*To*), peak gelatinization temperature (*Tp*), and heat of gelatinization (*ΔH*) were determined using the analysis tool available in the Universal Analysis software (TA instruments, New Castle, USA).

### In vitro digestion study

#### In vitro digestion procedure

The in vitro digestion was performed using the standard protocol reported by Brodkorb et al.^13^ with modifications. Briefly, the freshly cooked and prepared samples as described in the part of the preparation of starch-palmitic acid complex were immediately digested through a simulated oral phase, simulated gastric phase and simulated intestinal phase (SIP) with specific conditions (i.e., temperature, electrolytes, pH and enzymes). During the SIP, 100 μL of the samples were taken out from each tube at 0, 10, 20, 30, 45, 60, 90, 120, 150, and 180 min and mixed with 0.4 mL of ethanol to stop the digestion. Then the samples taken out at each time point were centrifuged at 4000 *g* for 15 min, and the supernatant was collected for glucose measurement.

#### Calculation of the percentage of the digested starch

The supernatant (100 µL) was combined with 0.5 mL of amyloglucosidase solution (27.16 U/mL) and incubated at 37 °C for 1 h. The tubes were then boiled for 5 min to inactivate the enzyme. After centrifugation at 4000 *g* for 15 min, the supernatant was collected for glucose measurement using GOPOD reagent (Megazyme Inc., Bray, Ireland). The digested amount of starch was calculated by the glucose quantity with a conversion factor of 0.9. The starch digestion data were fitted to a first-order model as previous described by Goñi, Garcia-Alonso, & Saura-Calixto (1997)^36^:

$${C}_{t}={C}_{0}+{C}_{\infty }\left(1-{e}^{(-kt)}\right)$$

Where C_t_, C_0_ and C_∞_ are the concentrations of the percentage of digested starch at time t, 0 and infinite time and *k* is a pseudo-first order rate constant. Box Lucas model as the nonlinear curve fit model in OriginPro 9 (OriginLab corporation, Northampton, MA, USA) was used for estimating *k* and C_∞_ value.

After digestion, the tubes were immediately centrifuged at 4000 g for 30 min at 4 °C. The supernatant was discarded and the sediment was stored at −20 °C for fermentation. The starch content in the remaining was measured using the Total Starch Assay Kit (AA/AMG) (Megazyme Inc., Bray, Ireland).

### In vitro batch fermentation

In vitro batch fermentation was performed using the method reported by Guo et al.^37^. Donors were based in the Netherlands, and fecal samples were collected on different days to perform the biological replicates. Fecal samples were quickly collected in a sterile container, in which an anaerobic atmosphere generation bag was added just before fecal collection, to obtain an anaerobic environment and stored at 4 °C before use. The subsequent process was completed within 2 h. All used equipment such as gloves, collection tubes, and swabs are sterilized to minimize the risk of contamination. Before fermentation, phosphate buffer for fecal inoculum (8.8 g/L K_2_HPO_4_, 6.8 g/L KH_2_PO_4_, 0.1 g/L sodium thioglycolate), fermentation medium (5.22 g/L K_2_HPO_4_, 16.32 g/L KH_2_PO_4_, 2 g/L NaHCO_3_, 2 g/L yeast extract, 2 g/L peptone, 1 g/L mucin, and 0.5 g/L L-cysteine HCL), and milli-Q water were sterilized by autoclave. Before use, the phosphate buffer was added with sodium dithionite (15 mg/L). The fresh feces (40.0 g) was combined with 200 mL anaerobic phosphate buffer and prepared by a Stomacher 400 circulator (Seward, UK) for 10 min at speed 300. The fecal suspension was centrifuged at 500 *g* for 2 min, and the supernatant was collected as the final fecal suspension. The digestion sediment was dispersed with 10 mL sterilized milli-Q water and added to penicillin bottles. The same amount of milli-Q water with or without palmitic acid was used as a control and blank sample. Then 21.5 mL medium and 3.5 mL fresh fecal sample was added into the penicillin bottles and covered by rubber caps. The bottles were flushed with nitrogen for 30 min for anaerobic conditions and then incubated at 37 °C on a rotating shaker (300 rpm). At each time point (0, 4, 24, and 48 h), 1.0 mL sample was taken out from the bottles with a sterilized syringe. All samples were stored at −20 °C for further analysis.

### Ethical approval

Briefly, donated fresh feces were collected from two healthy adults (27–28 years old, 18 < BMI < 23) who declared that they did not smoke and had not consumed antibiotics for 6 months. Healthy volunteers gave written consent for a single fecal donation, and their anonymity was granted. According to the Medical Ethical Advisory Committee of Wageningen University (METC-WU) guidelines, this research did not need ethical approval.

### SCFAs measurement and analysis

The samples collected at each time point were centrifuged at 9000 *g* (4 °C) for 5 min. The SCFAs content was measured using the method reported by Guo et al^37^.. For 500 µL supernatant, 250 µL internal standard (0.45 mg/mL 2-ethylbutyric acid in 0.3 M HCl and 0.9 M oxalic acid) was added for better SCFA separation and quantification. The samples were then injected into gas chromatography (GC-2014AFSC, Shimadzu, Hertogenbosch, the Netherlands), equipped with a Restek Stabilwax column 30 m × 0.32 mm × 0.5 µm (T Max 240 °C) (Restek, Santa Clara, CA, USA) and a flame-ionization detector (Shimadzu, Hertogenbosch, the Netherlands). The injection volume was 1.0 μL. The carrier gas was nitrogen, and the rate was 10 mL/min. The temperature profile starts at 100 °C, then increases at 10.8 °C/min to 180 °C, and is kept at 180 °C for 2 min. Then, it increased at 50.0 °C/min until 240 °C and kept at 240 °C for 2 min. The standards of acetic, propionic, butyric, valeric, iso-butyric and iso-valeric acids in concentrations of 0.01–0.45 mg/mL were prepared for identification and quantification.

The concentrations of acetic acid, propionic acid and butyric acid at 4 h, 24 h and 48 h were calculated and subjected to Time-series + one-factor analysis using MetaboAnalyst 5.0 online platform^38^. Select Auto-scaling (mean-centered and divided by the standard deviation of each variable) and Log transformation (based 10) for data normalization. The 3D Principal Component Analysis (PCA) plots and heatmaps were generated, and Correlation Analysis and ANOVA Simultaneous Component Analysis were processed.

### DNA extraction and high-throughput sequencing

Bacterial genomic DNA extraction from chosen samples after 48 h fermentation was performed using the Qiasymphony SP automated nucleic acid purification system (Qiagen, Germany). The extraction property and concentration were tested. 16S ribosomal RNA sequencing of the V3−V4 region was performed by PCR, using the forward primers (5’-CCTACGGGNGGCWGCAG-3’) and the reverse primer (5’-GACTACHVGGGTATCTAATCC-3’). Sequencing library was generated and quality was assessed by Quant-iT PicoGreen (Invitrogen, USA). Then the library was sequenced on Illumina MiSeq Platform (Illumina, San Diego, CA), which generated about 300 bp double-end reads with over 50,000 readings per sample and base mass was greater than 30.

### Data analysis

The 16S ribosomal RNA sequences were uploaded to NCBI as PRJNA1002494. Sequences were analyzed using Quantitative Insights into Microbial Ecology2 (QIIME2) software. DADA2 method generated feature sequences based on amplicon sequence variants (ASVs)^39^. Alpha diversity indexes (Shannon, Faith and Evenness index) were obtained using QIIME2 to indicate the gut microbiota composition in samples. The difference in microbial communities among samples was characterized by β-diversity calculated by principal coordinate analysis (PCoA) with weighted UniFrac analysis. The composition of each sample at the phylum level and the sequence number difference at the genus level were analyzed by alignment with Greengenes database. Bacterial metagenomics functions were predicted by phylogenetic investigation of communities by reconstruction of unobserved states (PICRUSt) on the 16S rRNA gene abundance data. Heatmap was performed to show diversity of gut microbiota composition and difference in predictive function among samples based on the most abundant ASVs (the relative content >1%) by R package. Integration of microbiome and metabolomics data was performed using Model-based Integration of Metabolite Observations and Species Abundances 2 (MIMOSA2), freely available at http://elbo-spice.cs.tau.ac.il/shiny/MIMOSA2shiny/^29^.

All tests were carried out in triplicates. One-way ANOVA and Duncan factorial scheme (significance level at *p* < 0.05) were performed for statistical analysis by SPSS 26 (SPSS Inc., Chicago, IL, USA).

### Reporting summary

Further information on research design is available in the Nature Research Reporting Summary linked to this article.

### Supplementary information

Supplemental Material

Reporting Summary

## Data Availability

The authors declare that all pertinent data that support this study have been included within the paper. The 16S ribosomal RNA sequences were uploaded to NCBI as PRJNA1002494. Raw data will be made available by corresponding authors upon request.
