# Supplementary material for: Lipid complexation reduces rice starch digestibility and boosts short-chain fatty acid production via gut microbiota
Source: NPJ Sci Food. 2023 Oct 18;7:56. doi: 10.1038/s41538-023-00230-1 (PMC10584848; doi:10.1038/s41538-023-00230-1)
Supplement: Supplementary file 1 — Supplemental Material [file 41538_2023_230_MOESM1_ESM.pdf]

Supplementary Information:

Lipid Complexation Reduces Rice Starch Digestibility and Boosts Short-Chain Fatty Acid Production via Gut Microbiota.

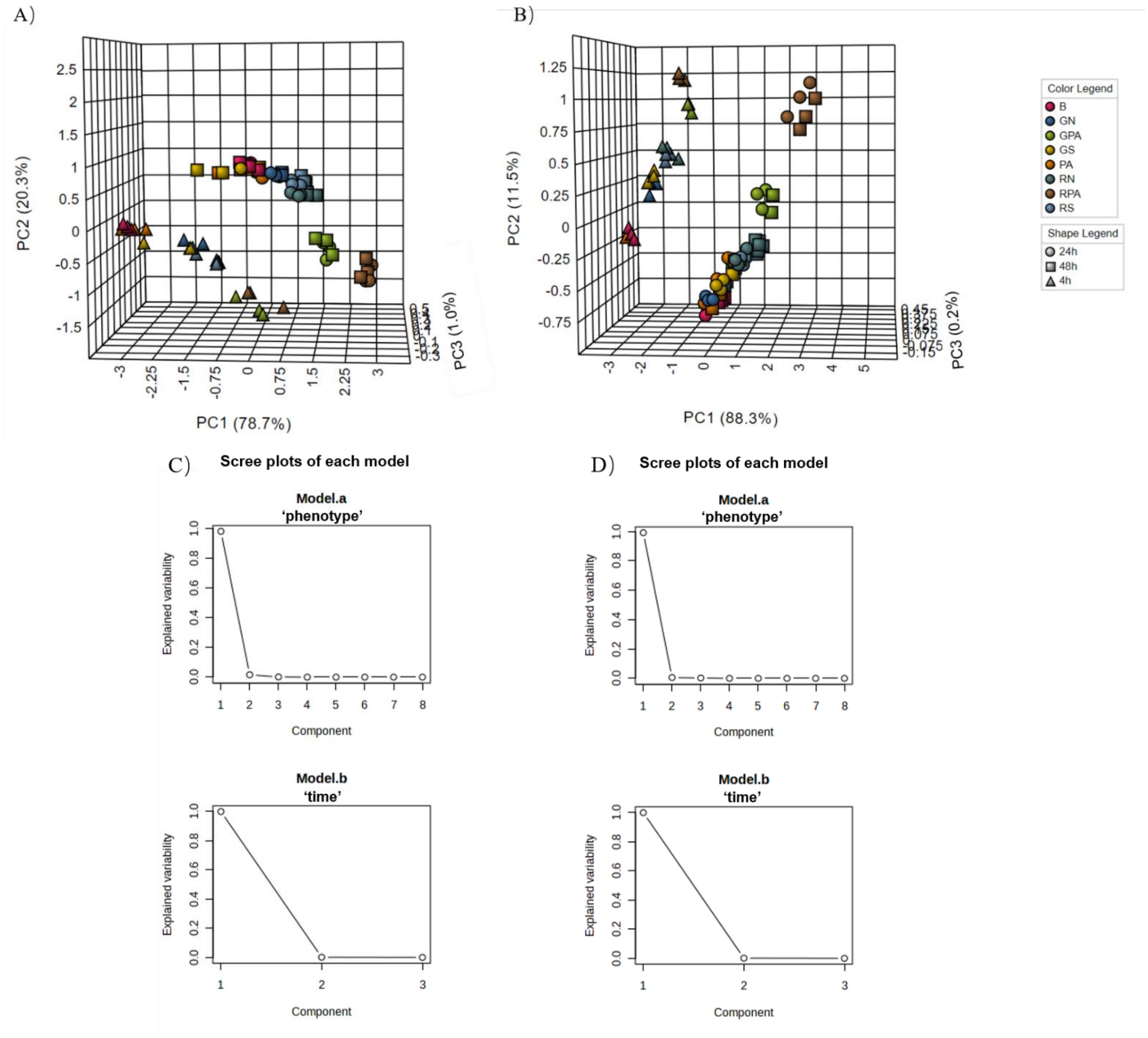

Supplementary Figure 1. The results performed by MetaboAnalyst 5.0. 3D PCA plots of donor1 A) and donor2 B). Scree plots of model 'phenotype' and 'time' in donor1 C) and donor2 D).

Supplementary Table 1. Remaining starch content after digestion of analyzed samples.

**Rice varieties**	**Sample name**	**Treatments**	**Remaining starch content after digestion (g/0.5 g)**
RS4	RN	native flour	0.15 ± 0.01[b]
RS4	RS	starch	0.14 ± 0.01[b]
RS4	RPA	starch + palmitic acid	0.21 ± 0.01[a]
GZ93	GN	native flour	0.12 ± 0.01[c]
GZ93	GS	starch	0.10 ± 0.01[c]
GZ93	GPA	starch + palmitic acid	0.16 ± 0.02[b]

Supplementary Table 2. Produced SCFAs of different samples when fermented for 4 h, 24 h, 48h. RN: RS4 native flour; RS: RS4 starch; RPA: RS4 starch complexed with palmitic acid; GN: GZ93 native flour; GS: GZ93 starch; GPA: GZ93 starch complexed with palmitic acid; PA, palmitic acid samples; B0, B4, B24, B48: blank sample fermented at 0, 4, 24, 48h. Different letters indicate significant differences at the 0.05 level.

	**Fermented time (h)**	**Sample**	**Acetic acid content (mM)**	**Propionic acid content (mM)**	**Butyric acid content (mM)**
Donor 1	0	B0	3.15 ±0.72 f	1.43 ±0.20 e	0.31 ±0.04 b
	4	GN	6.72 ±0.26 bc	5.83 ±0.36 bc	0.38 ±0.03 a
		GS	6.19 ±0.63 cd	5.33 ±0.68 bc	0.33 ±0.03 ab
		GPA	9.36 ±0.58 a	12.32 ±1.49 a	0.32 ±0.01 ab
		RN	7.74 ±0.05 b	7.08 ±0.23 b	0.33 ±0.01 ab
		RS	7.38 ±0.37 bc	6.94 ±0.75 b	0.35 ±0.02 ab
		RPA	9.89 ±0.87 a	12.05 ±2.06 a	0.32 ±0.03 ab
		PA	5.05 ±0.27 de	3.93 ±0.20 d	0.33 ±0.00 ab
		B4	4.91 ±0.09 e	3.93 ±0.13 d	0.38 ±0.00 ab
	24	GN	9.64 ±0.19 d	7.63 ±0.21 d	1.70 ±0.01 abc
		GS	9.23 ±0.83 de	7.12 ±0.76 d	1.60 ±0.12 c
		GPA	12.03 ±0.17 b	16.76 ±0.71 b	1.63 ±0.08 bc
		RN	10.67 ±0.24 c	10.54 ±0.35 c	1.79 ±0.04 ab
		RS	10.67 ±0.23 c	9.67 ±0.36 c	1.83 ±0.04 a
		RPA	15.19 ±0.22 a	24.71 ±0.62 a	1.69 ±0.12 abc
		PA	8.82 ±0.27 de	6.40 ±0.54 de	1.40 ±0.02 d
		B24	8.46 ±0.13 e	5.78 ±0.03 e	1.41 ±0.02 d
	48	GN	9.97 ±0.05 c	7.95 ±0.33 de	1.77 ±0.03 a
		GS	7.46 ±0.83 de	5.63 ±0.60 f	1.24 ±0.13 b
		GPA	11.74 ±0.39 b	16.29 ±1.27 b	1.77 ±0.02 a
		RN	11.37 ±0.32 b	9.90 ±0.38 c	1.85 ±0.05 a
		RS	11.33 ±0.27 bc	8.80 ±0.40 cd	1.96 ±0.01 a
		RPA	14.62 ±0.44 a	23.29 ±0.76 a	1.80 ±0.09 a
		PA	8.06 ±0.84 d	5.86 ±0.55 ef	1.34 ±0.20 b
		B48	8.43 ±0.41 cd	6.09 ±0.49 ef	1.48 ±0.06 b

Donor 2	0	B0	3.70 ±0.30 e	1.85 ±0.19 g	0.35 ±0.03 a
	4	GN	5.28 ±0.08 c	4.30 ±0.05 e	0.33 ±0.01 ab
		GS	5.40 ±0.14 c	4.65 ±0.20 de	0.33 ±0.01 ab
		GPA	7.33 ±0.05 a	6.59 ±0.43 b	0.32 ±0.01 abc
		RN	6.07 ±0.24 b	5.27 ±0.22 c	0.32 ±0.03 abc
		RS	5.97 ±0.10 b	5.06 ±0.10 cd	0.32 ±0.00 abc
		RPA	7.23 ±0.13 a	7.42 ±0.11 a	0.29 ±0.01 abc
		PA	4.13 ±0.08 d	2.85 ±0.10 f	0.29 ±0.01 c
		B4	4.31 ±0.10 d	3.07 ±0.11 f	0.31 ±0.03 bc
	24	GN	7.80 ±0.16 d	6.65 ±0.37 ef	1.30 ±0.07 cd
		GS	8.50 ±0.16 d	7.59 ±0.26 de	1.39 ±0.01 bc
		GPA	12.44 ±0.32 b	12.11 ±0.34 b	1.53 ±0.07 a
		RN	10.09 ±0.37 c	9.35 ±0.19 c	1.55 ±0.04 a
		RS	10.17 ±0.09 c	8.32 ±0.44 cd	1.53 ±0.04 a
		RPA	17.98 ±1.58 a	20.11 ±1.57 a	1.48 ±0.03 ab
		PA	8.03 ±0.82 d	5.92 ±0.79 ef	1.14 ±0.00 e
		B24	7.35 ±0.11 d	5.21 ±0.02 f	1.23 ±0.02 de
	48	GN	9.11 ±0.34 de	7.50 ±0.49 e	1.41 ±0.02 b
		GS	9.55 ±0.28 d	8.27 ±0.38 de	1.44 ±0.02 b
		GPA	13.39 ±0.13 b	13.45 ±0.14 b	1.73 ±0.08 a
		RN	11.52 ±0.13 c	10.48 ±0.09 c	1.74 ±0.08 a
		RS	11.60 ±0.23 c	9.51 ±0.43 cd	1.68 ±0.02 a
		RPA	18.77 ±1.19 a	20.77 ±1.31 a	1.75 ±0.00 a
		PA	8.16 ±0.51 e	5.96 ±0.35 f	1.32 ±0.01 b
		B48	8.20 ±0.18 e	5.79 ±0.05 f	1.36 ±0.02 b

Supplementary Table 3. α diversity index of the gut microbiota in analyzed fermented samples.

	Sample	Fermented time (h)	Feature sequence numbers	Shannon index	Faith index	Evenness index
Donor 1	B0	0	190 ± 8^{c}	5.5 ± 0.0^{a}	11.9 ± 0.6^{ab}	0.73 ± 0.00^{a}
	GN	48	198 ± 4^{bc}	5.2 ± 0.1^{b}	12.5 ± 1.2^{a}	0.68 ± 0.01^{c}
	GPA	48	170 ± 6^{d}	4.6 ± 0.1^{c}	12.1 ± 0.8^{ab}	0.63 ± 0.00^{d}
	RN	48	193 ± 4^{c}	5.2 ± 0.1^{b}	12.6 ± 0.7^{a}	0.68 ± 0.01^{c}
	RS	48	206 ± 7^{b}	5.2 ± 0.0^{b}	13.3 ± 1.5^{a}	0.67 ± 0.00^{c}
	RPA	48	143 ± 3^{e}	4.3 ± 0.0^{d}	10.1 ± 1.0^{b}	0.60 ± 0.00^{e}
	B48	48	228 ± 6^{a}	5.6 ± 0.0^{a}	12.4 ± 0.6^{a}	0.72 ± 0.00^{b}
Donor 2	B0	0	137 ± 1^{b}	4.8 ± 0.0^{b}	8.8 ± 0.4^{c}	0.68 ± 0.01^{b}
	GN	48	163 ± 5^{a}	5.2 ± 0.0^{a}	11.3 ± 0.8^{a}	0.70 ± 0.00^{a}
	GPA	48	125 ± 7^{c}	4.1 ± 0.0^{c}	9.8 ± 0.4^{abc}	0.59 ± 0.00^{c}
	RN	48	156 ± 5^{a}	4.9 ± 0.0^{b}	9.4 ± 0.3^{bc}	0.68 ± 0.00^{b}
	RS	48	153 ± 11^{a}	4.9 ± 0.1^{b}	10.5 ± 0.3^{abc}	0.67 ± 0.01^{b}
	RPA	48	111 ± 3^{d}	3.7 ± 0.1^{d}	11.1 ± 1.5^{ab}	0.55 ± 0.00^{d}
	B48	48	163 ± 1^{a}	5.2 ± 0.0^{a}	10.4 ± 0.3^{abc}	0.70 ± 0.00^{a}

Supplementary Table 4.The contributions of each taxon to metabolites variance based on MIMOSA2. Contribution results are only included for metabolites with model FDR q-value less than 0.01.

Compound	MetaboliteName	Rsq	VarDisp	ModelPVal	ModelPValFDRAdj	Slope	Intercept	Taxon	VarShare	NumSynthGenes	SynthGenes	NumDegGenes	DegGenes
C00033	Acetate	0.62106437	14.2069741	0.000112138	0.000112138	0.019623663	-2.89339242	*Enterobacter kobei*	0.621064369	1	R_CITACt	0	
C00163	propionate	0.54196712	14.4192631	0.00011493	0.000344791	-0.20248445	2.593299499	*Flavonifractor plautii ATCC 29863*	0.174892164	1	PPAt2	0	
C00163	propionate	0.54196712	14.4192631	0.00011493	0.000344791	-0.20248445	2.593299499	*Alistipes shahii WAL 8301*	0.137870555	1	PPAt2	0	
C00163	propionate	0.54196712	14.4192631	0.00011493	0.000344791	-0.20248445	2.593299499	*Blautia obeum ATCC 29174*	0.092150503	1	PPAt2	0	
C00163	propionate	0.54196712	14.4192631	0.00011493	0.000344791	-0.20248445	2.593299499	*Paraprevotella clara YIT 11840*	0.051331698	1	PPAt2	0	
C00163	propionate	0.54196712	14.4192631	0.00011493	0.000344791	-0.20248445	2.593299499	*Clostridium symbiosum ATCC 14940*	0.033035912	1	PPAt2	0	
C00163	propionate	0.54196712	14.4192631	0.00011493	0.000344791	-0.20248445	2.593299499	*Faecalibacterium prausnitzii M21 2*	0.02995414	1	PPAt2	0	
C00163	propionate	0.54196712	14.4192631	0.00011493	0.000344791	-0.20248445	2.593299499	*Alistipes onderdonkii DSM 19147*	0.028356913	1	PPAt2	0	
C00163	propionate	0.54196712	14.4192631	0.00011493	0.000344791	-0.20248445	2.593299499	*Clostridium hathewayi 12489931*	0.010388241	1	PPAt2	0	
C00163	propionate	0.54196712	14.4192631	0.00011493	0.000344791	-0.20248445	2.593299499	*Clostridium innocuum 2959*	0.004181604	1	PPAt2	0	
C00163	propionate	0.54196712	14.4192631	0.00011493	0.000344791	-0.20248445	2.593299499	*Actinomyces graevenitzii C83*	0.000658834	1	PPAt2	0	
C00163	propionate	0.54196712	14.4192631	0.00011493	0.000344791	-0.20248445	2.593299499	*Eubacterium hallii L2 7*	0.000599674	1	PPAt2	0	
C00163	propionate	0.54196712	14.4192631	0.00011493	0.000344791	-0.20248445	2.593299499	*Proteus mirabilis ATCC 29906*	0.000471597	0		1	PPAt2i
C00163	propionate	0.54196712	14.4192631	0.00011493	0.000344791	-0.20248445	2.593299499	*Rare/Low-abundance*	0.000443388	NA	NA	NA	NA
C00163	propionate	0.54196712	14.4192631	0.00011493	0.000344791	-0.20248445	2.593299499	*Alistipes finegoldii DSM 17242*	0.000228811	1	PPAt2	0	
C00163	propionate	0.54196712	14.4192631	0.00011493	0.000344791	-0.20248445	2.593299499	*Haemophilus parainfluenzae T3T1*	0.000131203	1	PPAt2	0	
C00163	propionate	0.54196712	14.4192631	0.00011493	0.000344791	-0.20248445	2.593299499	*Alistipes putredinis DSM 17216*	0	NA	NA	NA	NA
C00163	propionate	0.54196712	14.4192631	0.00011493	0.000344791	-0.20248445	2.593299499	*Bacteroides cellulosilyticus DSM 1483*	0	NA	NA	NA	NA
C00163	propionate	0.54196712	14.4192631	0.00011493	0.000344791	-0.20248445	2.593299499	*Bacteroides ovatus ATCC 8483*	0	NA	NA	NA	NA
C00163	propionate	0.54196712	14.4192631	0.00011493	0.000344791	-0.20248445	2.593299499	*Clostridium bolteae ATCC BAA 613*	0	NA	NA	NA	NA
C00163	propionate	0.54196712	14.4192631	0.00011493	0.000344791	-0.20248445	2.593299499	*Dorea longicatena DSM 13814*	0	NA	NA	NA	NA
C00163	propionate	0.54196712	14.4192631	0.00011493	0.000344791	-0.20248445	2.593299499	*Escherichia coli O157 H7 str Sakai*	0	1	PPAt2	1	PPAt2i
C00163	propionate	0.54196712	14.4192631	0.00011493	0.000344791	-0.20248445	2.593299499	*Ruminococcus obeum A2 162*	0	NA	NA	NA	NA
C00163	propionate	0.54196712	14.4192631	0.00011493	0.000344791	-0.20248445	2.593299499	*Gemella haemolysans ATCC 10379*	-0.00069179	1	PPAt2	0	
C00163	propionate	0.54196712	14.4192631	0.00011493	0.000344791	-0.20248445	2.593299499	*Streptococcus australis ATCC 700641*	-0.00081753	1	PPAt2	0	
C00163	propionate	0.54196712	14.4192631	0.00011493	0.000344791	-0.20248445	2.593299499	*Dorea formicigenerans ATCC 27755*	-0.00112998	1	PPAt2	0	
C00163	propionate	0.54196712	14.4192631	0.00011493	0.000344791	-0.20248445	2.593299499	*Clostridium celatum DSM 1785*	-0.00146534	1	PPAt2	0	
C00163	propionate	0.54196712	14.4192631	0.00011493	0.000344791	-0.20248445	2.593299499	*Eubacterium eligens ATCC 27750*	-0.00337299	1	PPAt2	0	
C00163	propionate	0.54196712	14.4192631	0.00011493	0.000344791	-0.20248445	2.593299499	*Streptococcus cristatus ATCC 51100*	-0.01525048	1	PPAt2	0	
C00246	butyrate	0.3835772	13.6438852	0.002989287	0.00448393	0.163380918	4.774554676	*Dorea longicatena DSM 13814*	0.229973607	0		1	BUTt2
C00246	butyrate	0.3835772	13.6438852	0.002989287	0.00448393	0.163380918	4.774554676	*Ruminococcus obeum A2 162*	0.197281016	0		1	BUTt2
C00246	butyrate	0.3835772	13.6438852	0.002989287	0.00448393	0.163380918	4.774554676	*Alistipes putredinis DSM 17216*	0.079925339	0		1	BUTt2
C00246	butyrate	0.3835772	13.6438852	0.002989287	0.00448393	0.163380918	4.774554676	*Blautia obeum ATCC 29174*	0.043830672	0		1	BUTt2
C00246	butyrate	0.3835772	13.6438852	0.002989287	0.00448393	0.163380918	4.774554676	*Escherichia coli O157 H7 str Sakai*	0.009826772	0		1	BUTt2
C00246	butyrate	0.3835772	13.6438852	0.002989287	0.00448393	0.163380918	4.774554676	*Actinomyces graevenitzii C83*	0	NA	NA	NA	NA
C00246	butyrate	0.3835772	13.6438852	0.002989287	0.00448393	0.163380918	4.774554676	*Alistipes finegoldii DSM 17242*	0	NA	NA	NA	NA
C00246	butyrate	0.3835772	13.6438852	0.002989287	0.00448393	0.163380918	4.774554676	*Alistipes onderdonkii DSM 19147*	0	NA	NA	NA	NA
C00246	butyrate	0.3835772	13.6438852	0.002989287	0.00448393	0.163380918	4.774554676	*Alistipes shahii WAL 8301*	0	NA	NA	NA	NA
C00246	butyrate	0.3835772	13.6438852	0.002989287	0.00448393	0.163380918	4.774554676	*Bacteroides cellulosilyticus DSM 1483*	0	NA	NA	NA	NA
C00246	butyrate	0.3835772	13.6438852	0.002989287	0.00448393	0.163380918	4.774554676	*Bacteroides ovatus ATCC 8483*	0	NA	NA	NA	NA
C00246	butyrate	0.3835772	13.6438852	0.002989287	0.00448393	0.163380918	4.774554676	*Clostridium celatum DSM 1785*	0	NA	NA	NA	NA
C00246	butyrate	0.3835772	13.6438852	0.002989287	0.00448393	0.163380918	4.774554676	*Clostridium hathewayi 12489931*	0	NA	NA	NA	NA
C00246	butyrate	0.3835772	13.6438852	0.002989287	0.00448393	0.163380918	4.774554676	*Clostridium innocuum 2959*	0	NA	NA	NA	NA
C00246	butyrate	0.3835772	13.6438852	0.002989287	0.00448393	0.163380918	4.774554676	*Clostridium symbiosum ATCC 14940*	0	NA	NA	NA	NA
C00246	butyrate	0.3835772	13.6438852	0.002989287	0.00448393	0.163380918	4.774554676	*Dorea formicigenerans ATCC 27755*	0	NA	NA	NA	NA
C00246	butyrate	0.3835772	13.6438852	0.002989287	0.00448393	0.163380918	4.774554676	*Eubacterium eligens ATCC 27750*	0	NA	NA	NA	NA
C00246	butyrate	0.3835772	13.6438852	0.002989287	0.00448393	0.163380918	4.774554676	*Eubacterium hallii L2 7*	0	NA	NA	NA	NA
C00246	butyrate	0.3835772	13.6438852	0.002989287	0.00448393	0.163380918	4.774554676	*Faecalibacterium prausnitzii M21 2*	0	NA	NA	NA	NA
C00246	butyrate	0.3835772	13.6438852	0.002989287	0.00448393	0.163380918	4.774554676	*Flavonifractor plautii ATCC 29863*	0	NA	NA	NA	NA
C00246	butyrate	0.3835772	13.6438852	0.002989287	0.00448393	0.163380918	4.774554676	*Gemella haemolysans ATCC 10379*	0	NA	NA	NA	NA
C00246	butyrate	0.3835772	13.6438852	0.002989287	0.00448393	0.163380918	4.774554676	*Haemophilus parainfluenzae T3T1*	0	NA	NA	NA	NA
C00246	butyrate	0.3835772	13.6438852	0.002989287	0.00448393	0.163380918	4.774554676	*Paraprevotella clara YIT 11840*	0	NA	NA	NA	NA
C00246	butyrate	0.3835772	13.6438852	0.002989287	0.00448393	0.163380918	4.774554676	*Proteus mirabilis ATCC 29906*	0	NA	NA	NA	NA
C00246	butyrate	0.3835772	13.6438852	0.002989287	0.00448393	0.163380918	4.774554676	*Streptococcus australis ATCC 700641*	0	NA	NA	NA	NA
C00246	butyrate	0.3835772	13.6438852	0.002989287	0.00448393	0.163380918	4.774554676	*Streptococcus cristatus ATCC 51100*	0	NA	NA	NA	NA
C00246	butyrate	0.3835772	13.6438852	0.002989287	0.00448393	0.163380918	4.774554676	*Rare/Low-abundance*	-0.00016759	NA	NA	NA	NA
C00246	butyrate	0.3835772	13.6438852	0.002989287	0.00448393	0.163380918	4.774554676	*Clostridium bolteae ATCC BAA 613*	-0.17709262	0		1	BUTt2

Supplementary Table 5.The mapped taxa data of all samples based on AGORA or EMBL_GEMs database (performed on MIMOSA2 website).

OUT (from AGORA databa	GN_1	GN_2	GN_3	RN_1	RN_2	RN_3	RS_1	RS_2	RS_3	GPA_1	GPA_2	GPA_3	RPA_1	RPA_2	RPA_3
Alistipes onderdonkii DSM 19147	243	203	144	605	859	1066	534	510	678	173	179	140	155	129	143
Clostridium innocuum 2959	15	12	17	11	9	5	11	14	8	7	7	10	4	0	0
Anaerostipes hadrus DSM 3319	347	275	288	232	211	228	148	319	198	146	224	145	161	141	130
Clostridium ramosum VPI 0427 DSM	0	4	7	9	2	4	3	0	0	2	5	0	0	0	2
Faecalibacterium prausnitzii M21 2	252	210	231	209	155	132	128	358	224	208	182	204	239	181	165
Enterobacter asburiae LF7a	5149	4697	4568	6357	7108	7357	6047	5634	6126	9665	9654	8999	13529	13058	12515
Eubacterium rectale ATCC 33656	494	474	633	6355	2457	3175	6022	9122	4210	658	779	726	568	456	426
Aeromonas caviae Ae398	0	0	0	0	0	0	0	0	0	0	0	3	0	0	0
Lachnospiraceae bacterium sp 8 1 57	193	114	120	133	118	161	106	188	128	66	69	81	13	9	4
Coprococcus catus GD 7	195	163	144	120	99	80	73	167	64	132	186	151	77	35	58
Alistipes finegoldii DSM 17242	7	0	0	0	6	5	0	0	0	0	0	0	0	0	0
Campylobacter jejuni jejuni ICDCCJ0	16	0	0	0	0	0	0	0	0	0	0	0	0	0	0
Dorea longicatena DSM 13814	1071	859	1101	845	947	827	676	698	1105	782	857	799	920	636	653
Fusobacterium ulcerans ATCC 49185	532	345	332	401	413	449	436	424	859	1164	1091	947	1281	1353	817
Faecalibacterium cf prausnitzii KLE1	51	40	59	136	100	119	89	163	121	0	0	0	0	0	0
Bifidobacterium catenulatum DSM 16	0	0	0	0	0	0	0	0	0	0	0	16	0	0	0
Bifidobacterium adolescentis ATCC 1	0	0	0	2	4	3	0	0	0	0	0	43	0	0	0
Bacteroides sp 4 1 36	628	600	533	938	917	953	607	758	595	830	857	797	1345	1222	1257
Bacteroides sp 20 3	729	634	517	832	697	753	723	1017	741	1478	1506	1551	3115	3118	2550
Enterococcus caccae ATCC BAA 124	6	0	3	0	0	0	0	0	0	0	0	0	0	0	0
Burkholderiales bacterium 1 1 47	3738	3586	3416	3326	3063	2881	2792	3943	2951	2128	2390	2223	2243	1961	1708
Bacteroides cellulosilyticus DSM 148	84	68	43	169	141	141	35	44	28	42	44	41	46	38	33
Bilophila wadsworthia 3 1 6	208	179	170	174	160	139	131	184	180	158	177	168	170	155	144
Veillonella sp 3 1 44	31	32	39	26	31	37	28	33	61	69	43	35	81	102	51
Dorea formicigenerans ATCC 27755	130	69	115	74	56	57	31	71	33	142	173	191	148	116	158
Anaerotruncus colihominis DSM 1724	15	11	13	0	8	5	5	12	0	0	0	0	0	0	0
Ruminococcus sp 5 1 39BFAA	846	578	576	462	456	462	417	577	443	408	413	367	166	132	118
Coprococcus comes ATCC 27758	1022	824	972	499	459	447	330	722	425	20	19	23	61	56	36
Eubacterium eligens ATCC 27750	261	177	205	218	249	251	217	204	313	276	326	234	319	321	251
Clostridium asparagiforme DSM 159	35	27	27	17	27	27	15	26	22	19	24	11	0	0	0
Bacteroides dorei DSM 17855	10784	9038	8527	10984	11077	11496	9740	11783	9901	15190	16017	15201	19097	18244	17623
Bacteroides stercoris ATCC 43183	4084	3784	3841	3681	3497	3640	2606	3979	2526	3019	3044	2948	3265	2986	2730
Ruminococcus bromii L2 63	38	30	28	961	466	947	1689	2825	1890	13	16	15	25	18	32
Streptococcus equinus ATCC 9812	0	0	0	0	0	4	0	0	0	0	0	0	0	0	0
Bacteroides coprocola M16 DSM 171	0	0	0	0	6	0	0	0	0	0	0	0	0	0	0
Eggerthella lenta DSM 2243	717	560	557	470	418	332	363	615	383	162	195	118	54	41	36
Collinsella aerofaciens ATCC 25986	0	0	0	0	2	1	0	0	0	0	0	13	0	0	0
Eubacterium ventriosum ATCC 27560	9	15	19	44	46	55	0	0	0	0	0	0	0	0	0
Actinomyces graevenitzii C83	3	0	3	0	0	2	2	0	0	0	0	3	0	0	0
Holdemania filiformis VPI J1 31B 1 D	66	48	49	38	38	28	39	50	31	5	9	4	0	0	0
Blautia obeum ATCC 29174	582	400	522	377	417	372	365	549	380	494	553	487	342	302	328
Bacteroides fragilis 3 1 12	1452	1331	1213	1462	1485	1581	1189	1699	1344	514	539	471	673	827	678
Eubacterium hallii DSM 3353	77	55	71	60	57	59	47	56	46	78	104	92	118	104	86
Eubacterium hallii L2 7	79	60	81	73	59	62	41	69	49	79	91	83	89	98	80
Clostridium bolteae ATCC BAA 613	150	145	132	110	146	135	99	141	154	320	372	353	803	902	756
Clostridium sp SS2 1	51	41	48	43	24	34	25	45	35	13	32	28	35	22	11
Proteus mirabilis ATCC 29906	0	0	0	0	0	0	0	0	0	0	5	9	3	0	0
Solobacterium moorei F0204	0	0	0	0	0	0	0	2	1	0	0	0	0	0	0
Streptococcus cristatus ATCC 51100	0	0	0	0	0	0	0	0	0	0	0	0	9	28	10
Eubacterium dolichum DSM 3991	19	6	9	5	9	8	6	19	6	0	0	0	0	0	0
Bacteroides nordii CL02T12C05	107	84	84	243	220	345	222	211	150	33	30	36	39	37	42
Streptococcus anginosus 1 2 62CV	0	0	0	0	0	0	0	3	0	0	0	0	0	0	0
Clostridium symbiosum WAL 14673	1093	849	1038	845	737	836	551	846	636	1255	1519	1317	1413	864	1064
Ruminococcus obeum A2 162	341	252	267	214	235	189	164	329	171	198	219	205	109	93	81
Streptococcus australis ATCC 700641	13	6	0	8	0	14	7	6	15	15	23	15	26	0	7
Clostridium hathewayi 12489931	19	19	12	15	16	11	12	16	0	0	0	0	0	0	0
Peptostreptococcus stomatis DSM 17	0	0	0	0	0	0	0	0	0	0	0	3	0	0	0
Lactobacillus ruminis ATCC 25644	0	0	0	2	0	0	0	0	0	0	0	0	0	0	0
Escherichia coli O157 H7 str Sakai	3129	2452	2188	1952	2100	2189	1769	2500	1933	3160	3144	2985	3222	2806	2973
Clostridium bartlettii DSM 16795	0	0	0	0	0	0	0	0	0	0	0	0	3	0	0
Bacteroides ovatus ATCC 8483	716	674	809	1548	1305	1399	1132	1154	671	162	175	158	311	291	366
Alistipes shahii WAL 8301	119	112	100	86	101	87	90	110	82	51	68	77	13	16	15
Citrobacter freundii ATCC 8090	0	0	0	0	0	0	0	0	0	0	0	0	2	0	0
Haemophilus parainfluenzae T3T1	5	0	0	2	7	5	3	4	0	0	0	0	0	0	0
Megasphaera elsdenii DSM 20460	0	0	0	0	0	0	0	0	0	0	0	27	0	0	0
Phascolarctobacterium succinatutens	0	0	0	0	0	0	0	0	0	0	0	0	0	2	0
Clostridium symbiosum ATCC 14940	32	30	20	27	30	21	19	28	12	16	17	16	4	0	0
Veillonella sp 6 1 27	31	36	42	34	34	32	30	11	49	57	45	48	77	100	50
Lactobacillus amylovorus GRL 1112	0	0	0	0	0	4	0	0	0	0	0	0	0	0	0
Subdoligranulum variabile DSM 1517	381	283	289	1682	1349	1411	1803	2262	1445	492	505	411	491	449	413
Fusobacterium varium ATCC 27725	78	55	51	69	68	77	79	36	129	173	193	149	206	224	131
Actinomyces cardiffensis F0333	0	0	0	0	0	0	0	0	0	0	3	0	0	2	0

Enterobacter aerogenes KCTC 2190	0	0	0	0	0	3	0	0	0	0	0	0	0	0	0
Anaerostipes caccae DSM 14662	0	0	2	0	0	0	0	0	0	0	0	0	0	0	0
Clostridium scindens ATCC 35704	41	28	28	28	22	30	25	31	21	13	13	11	0	0	0
Alistipes putredinis DSM 17216	149	117	114	56	67	73	104	109	101	9	10	0	0	2	0
Bacteroides finegoldii DSM 17565	0	2	0	0	0	0	0	3	0	0	0	0	0	0	0
Bacteroides faecis MAJ27	767	611	610	424	440	360	456	591	355	259	245	247	206	173	167
Clostridium celatum DSM 1785	0	0	0	0	0	0	0	2	0	2	2	0	0	3	2
Gemella haemolysans ATCC 10379	0	1	0	0	0	2	3	0	5	0	3	0	12	0	5
Clostridium indolis DSM 755	0	0	0	0	0	0	0	0	27	0	0	0	0	0	12
Alistipes indistinctus YIT 12060	19	16	17	18	12	16	19	22	10	18	25	23	33	25	30
Streptococcus thermophilus LMG 183	57	51	49	58	39	39	38	45	48	61	55	48	63	59	51
Eubacterium sulci ATCC 35585	0	0	0	0	0	0	0	0	0	0	0	0	0	0	1
Odoribacter splanchnicus 1651 6 DSM	91	79	76	66	61	67	65	114	67	36	23	28	26	27	24
Lactobacillus gasseri ATCC 33323	0	0	0	0	0	0	2	4	0	0	0	0	0	9	6
Cronobacter sakazakii ATCC BAA 89	0	0	0	0	0	0	1	0	0	0	2	0	0	0	0
Megamonas funiformis YIT 11815	0	0	0	0	2	0	0	0	0	0	0	107	0	0	0
Ruminococcus gnavus ATCC 29149	100	91	95	49	37	34	35	75	57	27	29	28	21	17	19
Clostridium leptum DSM 753	20	22	16	12	14	7	12	16	3	0	0	0	0	0	0
Erysipelotrichaceae bacterium 5 2 54	0	0	0	0	0	0	0	0	0	0	5	0	0	0	0
Eubacterium limosum KIST612	0	0	0	0	0	0	0	2	0	0	1	0	0	0	0
Flavonifractor plautii ATCC 29863	119	101	110	56	54	40	57	97	52	4	6	0	0	0	0
Streptococcus peroris ATCC 700780	0	0	0	0	1	0	0	0	0	0	0	0	0	0	0
Lactobacillus vaginalis ATCC 49540	0	0	0	8	0	0	0	0	0	0	0	0	0	0	0
Blautia hansenii VPI C7 24 DSM 205	94	70	137	13	22	9	15	15	15	6	10	7	19	11	7
Adlercreutzia equolifaciens DSM 194	36	22	19	22	20	18	14	29	17	3	19	13	4	10	5
Paraprevotella clara YIT 11840	114	124	113	102	61	46	21	64	12	0	0	0	0	0	0

**OUT (from EMBL_GEMs d**	**GN_1**	**GN_2**	**GN_3**	**RN_1**	**RN_2**	**RN_3**	**RS_1**	**RS_2**	**RS_3**	**GPA_1**	**GPA_2**	**GPA_3**	**RPA_1**	**RPA_2**	**RPA_3**
Clostridium innocuum 2959	15	12	17	11	9	5	11	14	8	7	7	10	4	0	0
Anaerostipes hadrus BPB5	398	316	336	275	235	262	173	364	233	159	256	173	196	163	141
Erysipelatoclostridium ramosum DSM	0	4	7	9	2	4	3	0	0	2	5	0	0	0	2
Allisonella histaminiformans DSM 15	1808	1401	1390	1304	1203	980	1054	1265	778	624	697	726	548	301	415
Siccibacter turicensis LMG 23730	74	66	46	59	64	83	60	112	59	58	44	66	73	57	51
Eubacterium rectale ATCC 33656	494	474	633	6349	2457	3175	6022	9113	4210	658	779	726	568	456	426
Ruminococcus torques ATCC 27756	21	11	19	16	15	15	11	17	14	0	0	0	0	0	0
Alistipes finegoldii DSM 17242	7	0	0	0	6	5	0	0	0	0	0	0	0	0	0
Dorea longicatena DSM 13814	1071	859	1101	845	947	827	676	698	1105	782	857	799	920	636	653
Fusobacterium ulcerans ATCC 49185	610	400	383	470	481	526	515	460	988	1337	1284	1096	1487	1577	948
Faecalibacterium prausnitzii A2 165	51	40	59	136	100	119	89	163	121	0	0	0	0	0	0
Bifidobacterium tsurumiense JCM 13	0	0	0	0	0	0	0	0	0	0	0	16	0	0	0
Phocea massiliensis Marseille P2769	38	35	34	25	26	29	29	43	37	15	13	15	2	0	0
Bifidobacterium longum NCC2705	0	0	0	2	4	3	0	0	0	0	0	43	0	0	0
Bacteroides uniformis ATCC 8492	628	600	533	938	917	953	607	758	595	830	857	797	1345	1222	1257
Ruthenibacterium lactatiformans 585	11	0	0	0	0	0	0	0	0	0	0	0	0	0	0
Parabacteroides distasonis ATCC 85	729	634	517	832	697	753	723	1017	741	1478	1506	1551	3115	3118	2550
Enterococcus pseudoavium NBRC 10	6	0	3	0	0	0	0	0	0	0	0	0	0	0	0
Parasutterella excrementihominis YIT	3738	3586	3416	3326	3063	2881	2792	3943	2951	2128	2390	2223	2243	1961	1708
Bacteroides cellulosilyticus WH2	84	68	43	169	141	141	35	44	28	42	44	41	46	38	33
Bilophila wadsworthia ATCC 49260	208	179	170	174	160	139	131	184	180	158	177	168	170	155	144
Dorea formicigenerans ATCC 27755	130	69	115	74	56	57	31	71	33	142	173	191	148	116	158
Anaerotruncus colihominis DSM 1724	15	11	13	0	8	5	5	12	0	0	0	0	0	0	0
Coprococcus comes ATCC 27758	1022	824	972	499	459	447	330	722	425	20	19	23	61	56	36
Eubacterium eligens ATCC 27750	261	177	205	218	249	251	217	204	313	276	326	234	319	321	251
Clostridium asparagiforme DSM 159	35	27	27	17	27	27	15	26	22	19	24	11	0	0	0
Bacteroides vulgatus ATCC 8482	10764	9023	8519	10967	11064	11483	9724	11765	9890	15180	15999	15184	19084	18222	17605
Bacteroides stercoris ATCC 43183	4084	3784	3841	3681	3497	3640	2606	3979	2526	3019	3044	2948	3265	2986	2730
Streptococcus equinus AG46	0	0	0	0	0	4	0	0	0	0	0	0	0	0	0
Bacteroides coprocola DSM 17136	0	0	0	0	6	0	0	0	0	0	0	0	0	0	0
Eggerthella lenta DSM 2243	717	560	557	470	418	332	363	615	383	162	195	118	54	41	36
bacterium LF 3	390	274	426	180	142	122	154	336	223	222	267	273	244	189	172
Collinsella aerofaciens ATCC 25986	0	0	0	0	2	1	0	0	0	0	0	13	0	0	0
Eubacterium ventriosum ATCC 27560	9	15	19	44	46	55	0	0	0	0	0	0	0	0	0
Actinomyces graevenitzii C83	3	0	3	0	0	2	2	0	0	0	0	3	0	0	0
Blautia obeum ATCC 29174	582	400	522	377	417	372	365	549	380	494	553	487	342	302	328
Bacteroides fragilis YCH46	1452	1331	1213	1462	1485	1581	1189	1699	1344	514	539	471	673	827	678
Eubacterium hallii DSM 3353	77	55	71	60	57	59	47	56	46	78	104	92	118	104	86
Clostridium citroniae WAL 17108	150	145	132	110	146	135	99	141	154	320	372	353	803	902	756
Proteus mirabilis HI4320	0	0	0	0	0	0	0	0	0	0	5	9	3	0	0
Streptococcus mitis B6	0	0	0	0	1	0	0	0	0	0	0	0	9	28	10
Enterobacter kobei	5075	4631	4522	6298	7044	7274	5987	5522	6067	9607	9610	8936	13456	13001	12464
Eubacterium dolichum DSM 3991	19	6	9	5	9	8	6	19	6	0	0	0	0	0	0
Bacteroides nordii CL02T12C05	107	84	84	243	220	345	222	211	150	33	30	36	39	37	42
Streptococcus anginosus C238	0	0	0	0	0	0	0	3	0	0	0	0	0	0	0
Streptococcus parasanguinis ATCC 1	13	6	0	8	0	14	7	6	15	15	23	15	26	0	7

Clostridium chauvoei JF4335	4	4	0	3	0	4	0	2	2	2	7	2	0	3	4
Romboutsia timonensis Marseille P32	4	0	0	3	3	2	2	3	4	2	3	0	0	2	4
Peptostreptococcus stomatis DSM 176	0	0	0	0	0	0	0	0	0	0	0	3	0	0	0
Lachnobacterium bovis DSM 14045	0	0	0	6	0	0	0	9	0	0	0	0	0	0	0
Lactobacillus ruminis ATCC 27782	0	0	0	2	0	0	0	0	0	0	0	0	0	0	0
Bacillus humi DSM 16318	3129	2452	2188	1952	2100	2189	1769	2500	1933	3160	3144	2985	3222	2806	2973
Faecalicatena fissicatena KCTC 1501	3	2	0	0	0	0	0	0	0	0	0	0	0	0	0
Intestinibacter bartlettii DSM 16795	0	0	0	0	0	0	0	0	0	0	0	0	3	0	0
Bacteroides ovatus ATCC 8483	716	674	809	1548	1305	1399	1132	1154	671	162	175	158	311	291	366
Alistipes shahii WAL 8301	119	112	100	86	101	87	90	110	82	51	68	77	13	16	15
Tumebacillus flagellatus GST4	0	0	0	0	0	0	0	0	0	0	0	0	2	0	0
Megasphaera elsdenii 14 14	0	0	0	0	0	0	0	0	0	0	0	27	0	0	0
Turicibacter sanguinis PC909	0	0	0	0	2	0	0	0	0	0	0	0	0	0	0
Phascolarctobacterium succinatutens	0	0	0	0	0	0	0	0	0	0	0	0	0	2	0
Clostridium symbiosum WAL 14163	32	30	20	27	30	21	19	28	12	16	17	16	4	0	0
Veillonella parvula DSM 2008	31	36	42	34	34	32	30	11	49	57	45	48	77	100	50
Lactobacillus amylovorus 30SC	0	0	0	0	0	4	0	0	0	0	0	0	0	0	0
Subdoligranulum variabile DSM 1517	381	283	289	1682	1349	1411	1803	2262	1445	492	505	411	491	449	413
Bittarella massiliensis GD6	6	1	3	6	2	2	0	5	3	0	0	0	0	0	0
Actinomyces odontolyticus ATCC 179	0	0	0	0	0	0	0	0	0	0	3	0	0	2	0
Serratia liquefaciens ATCC 27592	0	0	0	0	0	3	0	0	0	0	0	0	0	0	0
Anaerostipes caccae DSM 14662	0	0	2	0	0	0	0	0	0	0	0	0	0	0	0
Clostridium scindens ATCC 35704	41	28	28	28	22	30	25	31	21	13	13	11	0	0	0
Alistipes putredinis DSM 17216	149	117	114	56	67	73	104	109	101	9	10	0	0	2	0
Bacteroides thetaiotaomicron VPI 548	767	613	610	424	440	360	456	594	355	259	245	247	206	173	167
Gemella haemolysans ATCC 10379	0	1	0	0	0	2	3	0	5	0	3	0	12	0	5
Cloacibacillus porcorum CL 84	12	16	5	9	9	19	8	18	35	29	26	14	12	11	10
Alistipes indistinctus YIT 12060	19	16	17	18	12	16	19	22	10	18	25	23	33	25	30
Streptococcus thermophilus JIM 8232	57	51	49	58	39	39	38	45	48	61	55	48	63	59	51
Eubacterium sulci ATCC 35585	0	0	0	0	0	0	0	0	0	0	0	0	0	0	1
Odoribacter splanchnicus DSM 20712	91	79	76	66	61	67	65	114	67	36	23	28	26	27	24
Lactobacillus hominis DSM 23910 CR	0	0	0	0	0	0	2	4	0	0	0	0	0	9	6
Klebsiella pneumoniae subsp pneumoi	0	0	0	0	0	0	1	0	0	0	2	0	0	0	0
Megamonas funiformis YIT 11815	0	0	0	0	2	0	0	0	0	0	0	107	0	0	0
Ruminococcus gnavus AGR2154	100	91	95	49	37	34	35	75	57	27	29	28	21	17	19
Clostridium leptum DSM 753	20	22	16	12	14	7	12	16	3	0	0	0	0	0	0
Macrococcus canis KM45013	0	0	0	2	0	0	0	0	0	0	0	0	0	0	0
Eubacterium limosum ATCC 8486	0	0	0	0	0	0	0	2	0	0	1	0	0	0	0
Flavonifractor plautii YL31	119	101	110	56	54	40	57	97	52	4	6	0	0	0	0
Haemophilus haemoglobinophilus CC	5	0	0	2	0	0	3	0	0	0	0	0	0	0	0
Lactobacillus vaginalis DSM 5837 AT	0	0	0	8	0	0	0	0	0	0	0	0	0	0	0
Blautia hansenii DSM 20583	94	70	137	13	22	9	15	15	15	6	10	7	19	11	7
Adlercreutzia equolifaciens DSM 194	36	22	19	22	20	18	14	29	17	3	19	13	4	10	5
