# Supplementary material for: Lipid complexation reduces rice starch digestibility and boosts short-chain fatty acid production via gut microbiota
Source: NPJ Sci Food. 2023 Oct 18;7:56. doi: 10.1038/s41538-023-00230-1 (PMC10584848; doi:10.1038/s41538-023-00230-1)
Supplement: Supplementary file 2 — Reporting Summary [file 41538_2023_230_MOESM2_ESM.pdf]

nature portfolio

Corresponding author(s): Josep Rubert

Last updated by author(s): Jul 1, 2023

Reporting Summary

Nature Portfolio wishes to improve the reproducibility of the work that we publish. This form provides structure for consistency and transparency in reporting. For further information on Nature Portfolio policies, see our Editorial Policies and the Editorial Policy Checklist.

Statistics

For all statistical analyses, confirm that the following items are present in the figure legend, table legend, main text, or Methods section.

n/a	Confirmed	
☐	☒	The exact sample size (*n*) for each experimental group/condition, given as a discrete number and unit of measurement
☐	☒	A statement on whether measurements were taken from distinct samples or whether the same sample was measured repeatedly
☐	☒	The statistical test(s) used AND whether they are one- or two-sided<br>*Only common tests should be described solely by name; describe more complex techniques in the Methods section.*
☒	☐	A description of all covariates tested
☒	☐	A description of any assumptions or corrections, such as tests of normality and adjustment for multiple comparisons
☒	☐	A full description of the statistical parameters including central tendency (e.g. means) or other basic estimates (e.g. regression coefficient) AND variation (e.g. standard deviation) or associated estimates of uncertainty (e.g. confidence intervals)
☒	☐	For null hypothesis testing, the test statistic (e.g. *F*, *t*, *r*) with confidence intervals, effect sizes, degrees of freedom and *P* value noted<br>*Give P values as exact values whenever suitable.*
☒	☐	For Bayesian analysis, information on the choice of priors and Markov chain Monte Carlo settings
☒	☐	For hierarchical and complex designs, identification of the appropriate level for tests and full reporting of outcomes
☒	☐	Estimates of effect sizes (e.g. Cohen's *d*, Pearson's *r*), indicating how they were calculated

Our web collection on statistics for biologists contains articles on many of the points above.

Software and code

Policy information about availability of computer code

Data collection	Measurement of starch (Total Starch Assay Kit (AA/AMG), Megazyme Inc., Bray, Ireland)), protein (Dumas, thermo Quest NA 2100 Nitrogen and Protein Analyser, Interscience, Breda, the Netherlands), and lipid content (acid hydrolysis method (FNCPLSDM00018)). differential scanning calorimeter DSC Q200 (TA Instruments, New Castle, USA). SCFAs were quantified using a GC system (GC-2014AFSC, Shimadzu, Hertogenbosch, the Netherlands). Bacterial genomic DNA extraction was performed using the Qiasymphony SP automated nucleic acid purification system (Qiagen, Germany). 16S ribosomal RNA sequencing of the V3-V4 region was performed by PCR, using the forward primers (5′-CCTACGGGNGGCWGCAG-3′) and the reverse primer (5′-GACTACHVGGGTATCTAATCC-3′). Sequencing library was generated and quality was assessed by Quant-iT PicoGreen (Invitrogen, USA). Then the library was sequenced on Illumina MiSeq Platform (Illumina, San Diego, CA), which generated about 300 bp double-end reads with over 50000 readings per sample and base mass was greater than 30.
Data analysis	MetaboAnalyst 5.0. was employed to processes targeted metabolomics data. Sequences were analyzed using Quantitative Insights into Microbial Ecology2 (QIIME2) software. Bacterial metagenomics functions were predicted by phylogenetic investigation of communities by reconstruction of unobserved states (PICRUSt) on the 16S rRNA gene abundance data. Integration of microbiome and metabolomics data was performed using Model-based Integration of Metabolite Observations and Species Abundances 2 (MIMOSA2), freely available at http://elbo-spice.cs.tau.ac.il/shiny/MIMOSA2shiny

For manuscripts utilizing custom algorithms or software that are central to the research but not yet described in published literature, software must be made available to editors and reviewers. We strongly encourage code deposition in a community repository (e.g. GitHub). See the Nature Portfolio guidelines for submitting code & software for further information.

Data

Policy information about availability of data

All manuscripts must include a data availability statement. This statement should provide the following information, where applicable:

- Accession codes, unique identifiers, or web links for publicly available datasets
- A description of any restrictions on data availability
- For clinical datasets or third party data, please ensure that the statement adheres to our policy

The authors declare that all pertinent data that support this study have been included within the paper. Raw data will be made available by corresponding authors upon request.

Research involving human participants, their data, or biological material

Policy information about studies with human participants or human data. See also policy information about sex, gender (identity/presentation), and sexual orientation and race, ethnicity and racism.

Reporting on sex and gender	none
Reporting on race, ethnicity, or other socially relevant groupings	none
Population characteristics	Briefly, fresh feces were collected from two healthy adults (27-28 years old, 18 < BMI < 23).
Recruitment	Volunteers declared that they did not smoke and had not consumed antibiotics for 6 months.
Ethics oversight	*Identify the organization(s) that approved the study protocol.*

Note that full information on the approval of the study protocol must also be provided in the manuscript.

Field-specific reporting

Please select the one below that is the best fit for your research. If you are not sure, read the appropriate sections before making your selection.

☒ Life sciences ☐ Behavioural & social sciences ☐ Ecological, evolutionary & environmental sciences

For a reference copy of the document with all sections, see nature.com/documents/nr-reporting-summary-flat.pdf

Life sciences study design

All studies must disclose on these points even when the disclosure is negative.

Sample size	2 biological replicates and all analysis were performed in triplicate
Data exclusions	*Describe any data exclusions. If no data were excluded from the analyses, state so OR if data were excluded, describe the exclusions and the rationale behind them, indicating whether exclusion criteria were pre-established.*
Replication	all analysis were performed in triplicate
Randomization	GC and sequencing samples were randomly analized.
Blinding	*Describe whether the investigators were blinded to group allocation during data collection and/or analysis. If blinding was not possible, describe why OR explain why blinding was not relevant to your study.*

Reporting for specific materials, systems and methods

We require information from authors about some types of materials, experimental systems and methods used in many studies. Here, indicate whether each material, system or method listed is relevant to your study. If you are not sure if a list item applies to your research, read the appropriate section before selecting a response.

Materials & experimental systems

n/a	Involved in the study
☒	☐ Antibodies
☒	☐ Eukaryotic cell lines
☒	☐ Palaeontology and archaeology
☒	☐ Animals and other organisms
☒	☐ Clinical data
☒	☐ Dual use research of concern
☒	☐ Plants

Methods

n/a	Involved in the study
☒	☐ ChIP-seq
☒	☐ Flow cytometry
☒	☐ MRI-based neuroimaging
